# Eco-friendly silver nanoparticles from neem extracts: a dual approach to heavy metal sensing and antimicrobial applications

**DOI:** 10.1186/s40643-026-01011-w

**Published:** 2026-01-28

**Authors:** Samar O. Aljazzar, Abidemi Mercy Babatimehin, Oyebola Elizabeth Ogunbamowo, Moamen S. Refat, Lamia A. Albedair, Edwin Andrew Ofudje

**Affiliations:** 1 Department of Chemistry, College of Science, Princess Nourah bint Abdulrahman University, P.O. Box 84428, Riyadh 11671, Saudi Arabia; 2 Department of Chemical Science, Mountain Top University, Lagos-Ibadan Expressway, Mowe, Ogun State Nigeria; 3 Department of Biochemistry, Lagos State University, Ojo, Lagos Nigeria; 4 Department of Medical Biochemistry, Lagos State University College of Medicine, Ikeja, Lagos Nigeria; 5 Department of Chemistry, College of Science, Taif University, P.O. Box 11099, Taif 21944, Saudi Arabia

**Keywords:** Green synthesis, Silver nanoparticles, *Azadirachta indica*, Heavy metals, Colorimetric

## Abstract

This work investigates the green synthesis of silver nanoparticles (AgNPs) using mixed aqueous extracts of *Azadirachta indica* leaves and roots as natural reducing and stabilizing agents. The synthesis was optimized by varying extract concentration, pH, and temperature, and nanoparticle formation was confirmed by UV–Vis spectroscopy showing a characteristic surface plasmon resonance between 350 and 450 nm. Structural and morphological analyses {X-ray diffraction (XRD), scanning electron microscope (SEM), Fourier-Transform Infrared (FT-IR), particle size analysis} revealed predominantly crystalline, spherical AgNPs capped by phytochemicals like flavonoids, phenols, amide- and carbonyl-containing compounds. The phytochemical profile of the extract was further validated by Gas Chromatography-Mass Spectrometry (GC–MS) analysis. The biosynthesized AgNPs exhibited strong colorimetric sensing capability for heavy metals, showing noticeable spectral and visible color changes particularly in the presence of Hg^2^⁺, Pb^2^⁺, and Cd^2^⁺ ions. Antibacterial evaluation indicated significant inhibitory activity against *Staphylococcus aureus* (33 ± 0.13 mm) and *Escherichia coli* (45 ± 0.21 mm), outperforming standard gentamycin controls. These findings highlight neem-derived AgNPs as low-cost, eco-friendly nanomaterials with dual applications in environmental monitoring of heavy metals and antimicrobial therapy.

**Graphical abstract:**

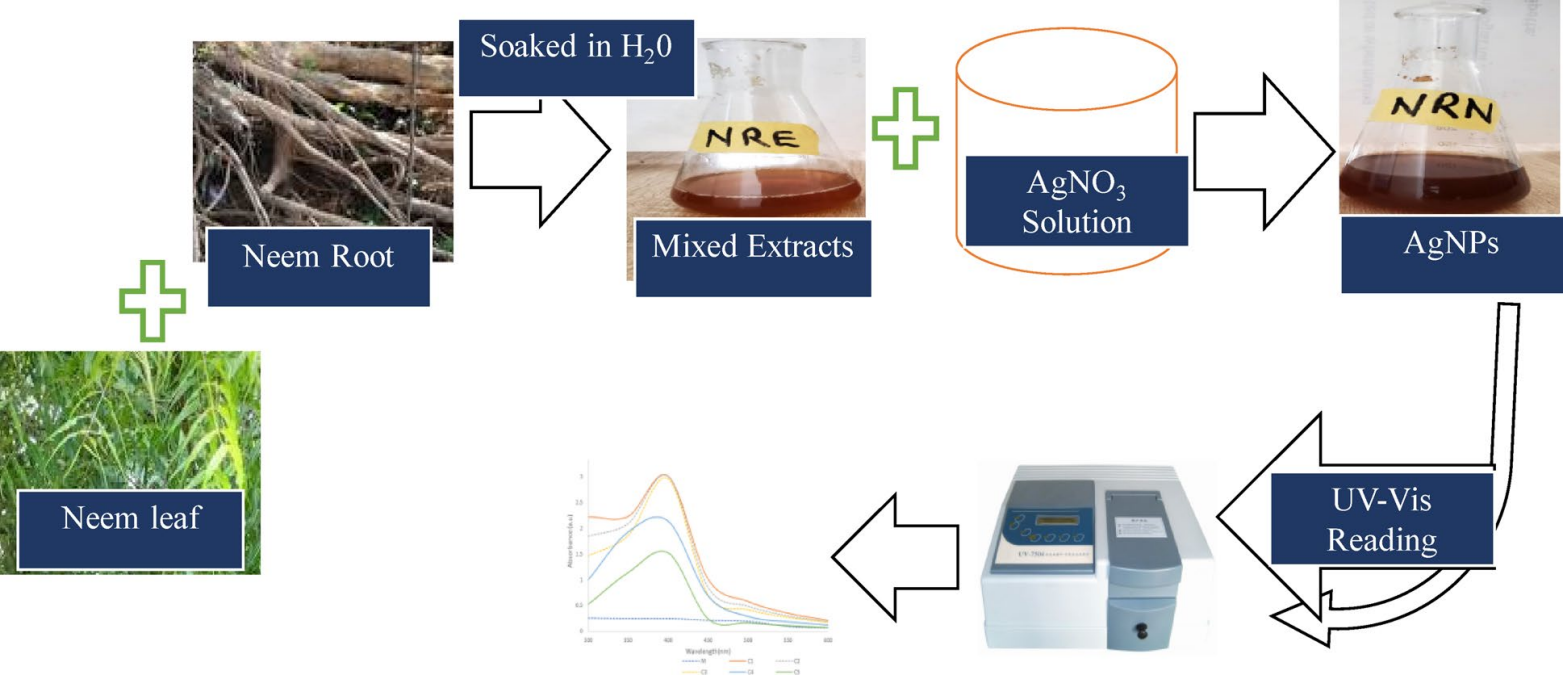

## Introduction

Heavy metals, typically characterized by high atomic weight, include both essential and toxic elements such as cadmium (Cd), chromium (Cr), lead (Pb), mercury (Hg), copper (Cu), and zinc (Zn)(Balali-Mood et al. 2021) . These elements exhibit dual roles in biological systems: some are vital micronutrients, while others pose significant toxicity risks even at low concentrations (Adegoke et al. 2014; Ofudje et al. 2020; Tilako et al. 2020). Increasing industrial and agricultural activities, driven by global population growth, have intensified the release of heavy metal-laden effluents into the environment (Adegoke et al. 2014). This contamination progressively degrading ecosystems, impairing their ability to sustain biodiversity and provide essential ecological services (Giacoma et al. 2021; Tosini et al. 2023).

Exposure to heavy metals is associated with serious health effects, including carcinogenic, genotoxic, teratogenic, and mutagenic outcomes (Ali et al. 2013; Ofudje et al. 2023). While some heavy metals are necessary for biological functions, excessive exposure can damage cellular structures in both plants and animals (Babatimehin et al. 2025a). Industrial reliance on these elements further complicates containment efforts, often overshadowing their beneficial uses with substantial environmental and public health risks (Balali-Mood et al. 2021). For example, lead exposure is linked to intellectual deficits in children (Mason et al. 2014), mercury poisoning is connected to *Minamata* disease, and cadmium exposure is associated with *itai-itai* disease (Inaba et al. 2005). Additional health risks include neurotoxicity, hepatotoxicity, and nephrotoxicity (Baruah et al. 2016).

Environmental contamination by heavy metals arises from both anthropogenic activities like mining, agriculture, manufacturing, and natural processes like weathering (De Silva et al. 2025; Laoye et al. 2025; Mohammad et al. 2025). Pollution sources are classified as point sources (factory discharge) or non-point sources (agricultural runoff, urban stormwater), with the latter being particularly challenging to monitor and mitigate due to their diffuse origin (Laoye et al. 2025; Mohammad et al. 2025). Accurate detection and quantification of heavy metals are essential for implementing effective remediation strategies in soil and water systems (Aziz et al. 2023; Xu et al. 2024). Proper environmental management of these pollutants is critical due to their persistence and toxicity. 

Microorganisms, on the other hand, play a crucial role in human life with both beneficial and harmful impacts. On the positive side, they are essential in food production offering services such as fermentation (for yogurt, bread, and cheese), agriculture (biological nitrogen fixation), medicine (antibiotics, vaccines, and enzymes), and management of the environment (bioremediation and biodegradation). On the contrary, some of these microorganisms can cause diseases in animals, humans, and plants, leading to illnesses such as malaria (protozoa), tuberculosis, cholera, and fungal infections. Wastewater often contains pathogenic microorganisms from animal and human waste, hospital effluents, and industrial discharges. If not removed, these microbes can spread diseases and illness through contaminated water sources. Thus, effective wastewater treatment ensures public health safety, protects aquatic ecosystems, prevents outbreaks, and provides cleaner water for reuse.

Conventional detection methods such as Inductively Coupled Plasma Mass Spectrometry (ICP-MS), Inductively Coupled Plasma Optical Emission Spectrometry (ICP-OES and Atomic Absorption Spectroscopy (AAS) offer high sensitivity and specificity (Chandraker et al. 2019; Xu et al. 2024). However, their operational complexity, prolonged analysis time, and high cost often restrict widespread or field-based application (Chandraker et al. 2019). Nanotechnology has emerged as a promising alternative, leveraging materials engineered at the nanometer scale (1–100 nm) where unique size-dependent properties enhance reactivity and functionality (Chandramohan et al. 2016; Chen et al. 2019). These nanomaterials exhibit tunable optical properties, high surface area, and potential reusability, making them suitable for environmental sensing and remediation (Dakal et al. 2016; Babatimehin et al. 2025b).

Green synthesis of nanoparticles using plant extracts provides an eco-friendly route that utilizes phytochemicals as reducing and stabilizing agents, avoiding toxic reagents and energy-intensive processes (Fahimmunisha et al. 2020; Dawadi et al. 2021; Ekundayo et al. 2024). Silver nanoparticles (AgNPs), in particular, exhibit strong surface plasmon resonance and high sensitivity, enabling colorimetric detection of heavy metal ions at low concentrations (Dawadi et al. 2021; Babatimehin et al. 2025a). Green-synthesized silver-based nanoparticles often incorporate biomolecules from the biological extract like lipids, proteins, enzymes, nucleic acids that act as reducing and stabilizing/capping agents. These capping agents naturally derived may remain associated with the nanoparticle surface, and can impact the biological property of the particles, potentially facilitating antimicrobial activity through synergistic interactions (Lashgarian et al. 2021; Mirzaei et al. 2021).

Silver-based nanoparticles are widely documented to possess broad-spectrum antimicrobial activity against fungi, Gram-positive and Gram-negative bacteria, and biofilm-forming strains. Different biological studies have exhibited measurable zones of inhibition and dose-dependent antibacterial effects for algal- or plant-mediated Ag nanoparticles, demonstrating their potency as antimicrobial agents (Gholami et al. 2018; Mirzaei et al. 2021). For instance, AgNPs obtained using *Thymus kotschyanus* revealed clear antibacterial activity by the disc diffusion approach against both Gram-positive and Gram-negative bacteria (Gholami et al. 2018). The AgNPs antimicrobial mechanisms are complementary and multifactorial and some of the modes of action already reported are (i) release of Ag⁺ ions that bind phosphate and thiol groups on DNA and enzymes, disrupting metabolic processes and replication; (ii) generation of reactive oxygen species (ROS) that oxidatively destroy membrane lipids, nucleic acids and proteins; and (iii) direct nanoparticle–cell membrane interaction that enhances membrane permeability and causes structural damage (Lashgarian et al. 2021; Mirzaei et al. 2021). These effects can combine together to inactivate enzymes, inhibit respiration, and ultimately lead to cell damage.

The green synthesis approach further enhances their applicability by reducing toxicity and production costs. This study utilizes neem (*Azadirachta indica*) mixed extracts of leaves and root for the synthesis of AgNPs, optimizing key parameters like temperature, pH, and precursor concentration to achieve stable and effective nanoparticles. The nanoparticles produced were characterized using UV–Visible Spectroscopy, Fourier-Transform Infrared Spectroscopy (FT-IR), X-Ray Diffraction (XRD), and Scanning Electron Microscopy with Energy-Dispersive X-ray Spectroscopy (SEM/EDX). Additionally, the sensing ability towards selected metal ions and the antimicrobial efficacy of the AgNPs were assessed against both gram-positive and gram-negative microorganisms.

## Materials and methods

### Materials

Sodium hydroxide (NaOH, 98%), hydrochloric acid (HCl, 37%), Silver nitrate (AgNO₃ 99% purity), and other reagents used such as PbCl_2_, CuSO_4_, FeSO_4_, NaCl, LiCl, CoCl_2,_ and CdCl_2_ were of high purity and were purchased from Sigma Aldrich in Cape Town, South Africa. All glass wares were cleaned with distilled and deionized water and dried in an oven.

### Preparation of extract from plant sources

Leaves and roots of *Azadirichta indica* (neem) were collected from Lagos State University Zoological Garden, Nigeria (GPS N-06° 28.214 E-003 11.864). The authentication of the plant species was done by subject experts at the Botany Department, Lagos State University, Nigeria. The collected leaves and roots were washed repeatedly with distilled water to remove dirt and dried at room temperature for 15 days. The samples were reduced into smaller sizes and pulverized into its powdery forms and mixed together in same proportion. Thereafter, 5 g of the mixed powdered *Azadirachta indica* leaf and root was soaked in 100 mL of H₂O for 24 h. Subsequently, the mixture was sieved and the obtained extract solution was kept and thereafter used for nanoparticle synthesis.

### Phytochemical analysis of plant extract

Preliminary phytochemical screening of the neem extract was carried out following the approaches previously outlined by Babatimehin et al. (2025a) to identify the major bioactive constituents responsible for reduction and stabilization during nanoparticle synthesis. The phytochemical composition of the plant extract was analyzed by Gas Chromatography-Mass Spectrometry (GC–MS). The analysis was performed using an Agilent Technologies 7820A gas chromatograph (USA) coupled to a 5975C inert mass selective detector (equipped with a triple-axis detector) and an electron ionisation (EI) source. The mass spectrometer was operated in full scan mode, covering a mass-to-charge (m/z) range of 30 to 550 atomic mass units (amu) with a scan rate of 2.62 s per scan.

### Synthesis of silver nanoparticles

The silver nanoparticles (AgNPs) were synthesized using a modified green synthesis approach adapted from the method of Babatimehin et al. (2025b). Silver nitrate solution was prepared at a controlled concentration of 1 mM AgNO₃ using analytical-grade AgNO₃ dissolved in distilled water. For nanoparticle synthesis, 10 mL of the plant mixed extract was added dropwise to 90 mL of the 1 mM AgNO₃ solution under continuous stirring at room temperature. while the pH adjustment was done using 0.1 M NaOH or HCl. The extract-to-AgNO₃ ratio (1:9 v/v) was kept constant to ensure uniform reduction conditions across all synthesis runs. The selected conditions (extract volume, AgNO₃ concentration, and mixing ratio) were based on preliminary trials where different extract volumes (5–20 mL) and AgNO₃ concentrations (0.5–2 mM) were tested for nanoparticle formation. To determine the optimal synthesis conditions, the concentration of the plant extract was systematically varied from 6.25 to 100 mg/mL. The reaction mixture was incubated at temperatures ranging from 25 to 100 °C under constant agitation at 120 rpm using an orbital shaker. The formation of AgNPs was monitored after one hour by measuring the absorbance of the solution with a UV–Vis spectrophotometer (Model SM 7504, Shanghai, China), scanning wavelengths from 300 to 600 nm. This entire procedure was subsequently repeated while independently varying the like of parameters such as pH, incubation temperature, and extract concentration so as to comprehensively evaluate their individual effects on nanoparticle synthesis and stability.

### Characterizations

The synthesized silver nanoparticles (AgNPs) were comprehensively characterized using a suite of analytical techniques.

*Optical properties* The formation and stability of the AgNPs were monitored using a UV–Visible spectrophotometer (Model SM 7504, Shanghai, China). This instrument was also employed for subsequent sensing applications.

*Morphological and elemental composition analysis* The surface morphology and shape of the AgNPs were examined using a Hitachi S-3000H (Japan) scanning electron microscope (SEM), operating at an accelerating voltage of 15 kV. The elemental composition and chemical analysis of the AgNPs were confirmed by energy-dispersive X-ray spectroscopy (EDX) attached to the SEM.

*Crystalline structure* The crystallographic structure of the nanoparticles was determined by X-ray diffraction (XRD) using a Rigaku Miniflex 600, diffractometer (JAPAN). Measurements were recorded in the 2θ range of 10° to 70°. The resulting diffraction peaks were indexed by comparison with the standard reference data from the Joint Committee on Powder Diffraction Standards (JCPDS).

*Size distribution* The particle size distribution of the prepared AgNPs was analyzed using a Nanotrac particle size analyzer (USA), with data processed via the accompanying Microtrac FLEX software (version 10.5.2).

*Functional group analysis* Fourier-Transform Infrared (FT-IR) spectroscopy was performed on the synthesized AgNPs to identify the functional groups responsible for reduction and capping. Spectra were acquired using a Thermo Fisher Scientific CARY 630 instrument (USA). Samples were prepared using the KBr pellet method, wherein 1% (w/w) of the sample was homogenized with 99% KBr and compressed into a 2 mm diameter pellet. All spectra were collected in the mid-infrared range of 400–4000 cm^−1^ at a resolution of 4 cm^−1^, averaging 64 scans per spectrum.

### Applications

#### Metal ions recognition ability of the synthesized AgNPs

The capacity of the biosynthesised silver nanoparticles (AgNPs) to act as a colorimetric sensor for aqueous metal ions was evaluated according to an established protocol (Babatimehin et al. 2025a). In a standard assay, 1 mL of the synthesized AgNP solution was mixed with 1 mL of a 100 µM aqueous solution of a target metal ion. This study included ions from the following salts: NaCl, PbCl₂, FeSO₄, CuSO₄, CdCl₂, ZnCl₂, and NiCl₂. The reaction mixtures were maintained at 35 °C. The sensing performance was assessed by monitoring two key parameters: visible colorimetric change in the solution and the corresponding shift in the absorption intensity, as measured by UV–Vis spectroscopy. All measurements were performed in triplicate (n = 3), and data are presented as mean ± standard deviation.

#### Antibacterial applications of the synthesized AgNPs

The antibacterial efficacy of the biosynthesized silver nanoparticles (AgNPs) was evaluated against *Staphylococcus aureus* (*S. aureus*) and *Escherichia coli* (*E. coli*) using a well diffusion assay, following established methodologies (Francine et al. 2015; Babatimehin et al. 2025a). Mueller–Hinton agar plates were inoculated by spreading 0.2 mL of a standardized bacterial inoculum (adjusted to 0.5 McFarland standard) uniformly across the surface using a sterile Pasteur pipette and a hockey stick-shaped glass spreader. Subsequently, two wells, each 6 mm in diameter, were aseptically bored into the agar using a flame-sterilized cork borer. The plates were allowed to rest for 5 min before the test extracts were introduced into the wells. To facilitate compound diffusion, the plates were held at room temperature for 30 min.

#### Test for minimum inhibitory concentrations (MIC)

The minimum inhibitory concentration (MIC), defined as the lowest concentration of AgNPs that prevented visible growth of the test microorganisms, was determined using a broth dilution method (Francine et al. 2015; Babatimehin et al. 2025a). A stock solution of the AgNPs was prepared at 500 µg/mL by adding 2 mL of the nanoparticle suspension to 4 mL of peptone water. This stock was then serially diluted into two-fold to obtain a concentration range of 500, 250, 125, 62.50, 31.25, 15.62, 7.81, and 3.90 µg/mL. Following dilution, each tube was inoculated with three drops of a standardized broth culture of the test organism (*S. aureus* or *E. coli*). The inoculated tubes were subsequently incubated at 37 °C for 24 h. After the incubation period, the MIC was identified as the tube with the lowest concentration of AgNPs that showed no visible turbidity, indicating complete inhibition of microbial growth. All antimicrobial assays (disc diffusion) were conducted in triplicate (n = 3) and for each concentration of AgNPs, three independent plates were prepared and measured.

#### Test for minimum bactericidal concentrations (MBC)

Following the MIC incubation period, a sub-culturing technique was employed to determine the minimum bactericidal concentration (MBC). A sample from each tube that showed no visible growth was aseptically transferred onto sterile nutrient agar plates. These plates were then incubated at 37 °C for 24 h. The MBC was defined as the lowest concentration of AgNPs at which no bacterial colonies formed on the nutrient agar, indicating that the treatment was bactericidal and had killed the inoculum rather than merely inhibiting its growth.

#### Cytotoxicity evaluation (MTT assay)

To assess the cytocompatibility of the AgNPs, the NIH3T3 mouse fibroblast cell line (NCCS, Pune) was used. Cell viability was analyzed using the MTT ([3-(4, 5-dimethylthiazol-2-yl)-2, 5-diphenyl-tetrazolium bromide]) assay as previously described Liaqat et al. (2022) and Tin-Oo et al. (2007). In brief, NIH3T3 cells (10,000 per well) were plated in a 24-well plate and allowed to adhere for 24 h. After attachment, the medium was replaced with Dulbecco’s Modified Eagle Medium (DMEM) containing diverse concentrations of AgNPs, while cells exposed only to Ag in DMEM served as the control group. Following a 48-h incubation period, the medium was removed, and the cells were rinsed with Phosphate-Buffered Saline (PBS) before being exposed to an MTT solution. The cells were then incubated for 4 h at 37 °C in a CO₂-controlled environment. After incubation, the MTT solution was discarded, the cells were washed with PBS, and dimethyl sulfoxide (100 µL) was added to dissolve the resulting formazan crystals. The absorbance of the resulting solution was measured at 570 nm, and cell viability in the treated samples was compared to the control.

#### Analytical validation and selectivity tests

The calibration validation was determined using the modified method previous reported by Sahu et al. (2024), Rossi et al. (2021) and Roto et al. (2019). Briefly, the calibration series were made by mixing 1 mL AgNP suspension with 1 mL of metal-ion solutions at known concentrations (0, 5, 10, 15, 25, 50, 75, 100 µ/L) and UV–Vis absorbance were measured at the sensor λmax (λ ≈ 400 nm). Each determination point was measured in triplicate (n = 3) and the mean ± standard deviation recorded. The blank (0 µ/L metal; AgNP + solvent only) was measured ten times to determine the instrument/sample baseline noise (σblank). Calibration curves (absorbance vs concentration) were described by the linear least square equation: y = mx + c, where x represents the concentration of metal ions, y represents the absorbance value, the slope as m, and intercept denoted as c. The limits of detection (LOD) and quantification (LOQ) were computed using LOD = 3.3·σblank/m and LOQ = 10·σblank/m. All experiments were performed at 35 °C, consistent with the sensing assay described in the manuscript. For the selectivity experiment, different solutions of ions K^+^, Na^+^, Ca^2+^, Cd^2+^, Hg^2+^, and Pb^2+^ with a concentration of 100 µg/L were prepared and placed in separate test tubes. Then, AgNPs were introduce to each solution and the effect of competing were computed by measuring the UV–Vis spectrum for each solution in the range of 300–600 nm.

## Results and discussion

### Phytochemical analysis

The qualitative and quantitative tests which were preliminary carried out to determine the phytochemicals present in the plant extract are presented in Tables 1 and 2 respectively. The phytochemical screening of the plant extract confirmed the presence of various secondary metabolites, including sugars, triterpenes, amino acids, and phenols (Table 1). The absence of phlebotannins was also noted, a finding consistent with the report by Dash et al. (2017). Quantitative analysis (Table 2) confirmed that phenols were found to be the most abundant compound (91.40 mg/mL), followed by tannins (88.65 mg/mL), and flavonoids (74.48 mg/mL). The extract also contained substantial quantities of alkaloids (38.20 mg/mL), reducing sugars (18.80 mg/mL) and cardiac glycosides (17.77 mg/mL). These bioactive compounds, particularly the phenols and flavonoids, are directly responsible for the bioreduction of silver ions (Ag⁺) to elemental silver nanoparticles (Ag⁰) and subsequently act as capping and stabilising agents (Dhir et al. 2023; Babatimehin et al. 2025b). The effectiveness of these plant-derived capping agents was evidenced by the stability of the AgNPs in colloidal solution, which showed no signs of agglomeration for at least six hours post-synthesis.

**Table 1:** Qualitative analysis of neem leaf and root mixed extract

Tannin	Phenol	Phlobatannins	Alkaloid	Saponin	Flavonoid	Steroid	Anthraquinone	Terpenoid	Cardiac glycoside	Reducing sugar
+	+	+	+	+	+	+	+	+	+	+

**Table 2:** Quantitative analysis of neem leaf and root mixed extract (mg/mL)

	Tannin	Phenol	Saponin	Alkaloid	Reducing sugar	Cardiac glycoside	Flavonoid
	88.65	91.40	27.64	38.20	18.80	17.77	74.48

### GC–MS analysis

**Fig. 1:**
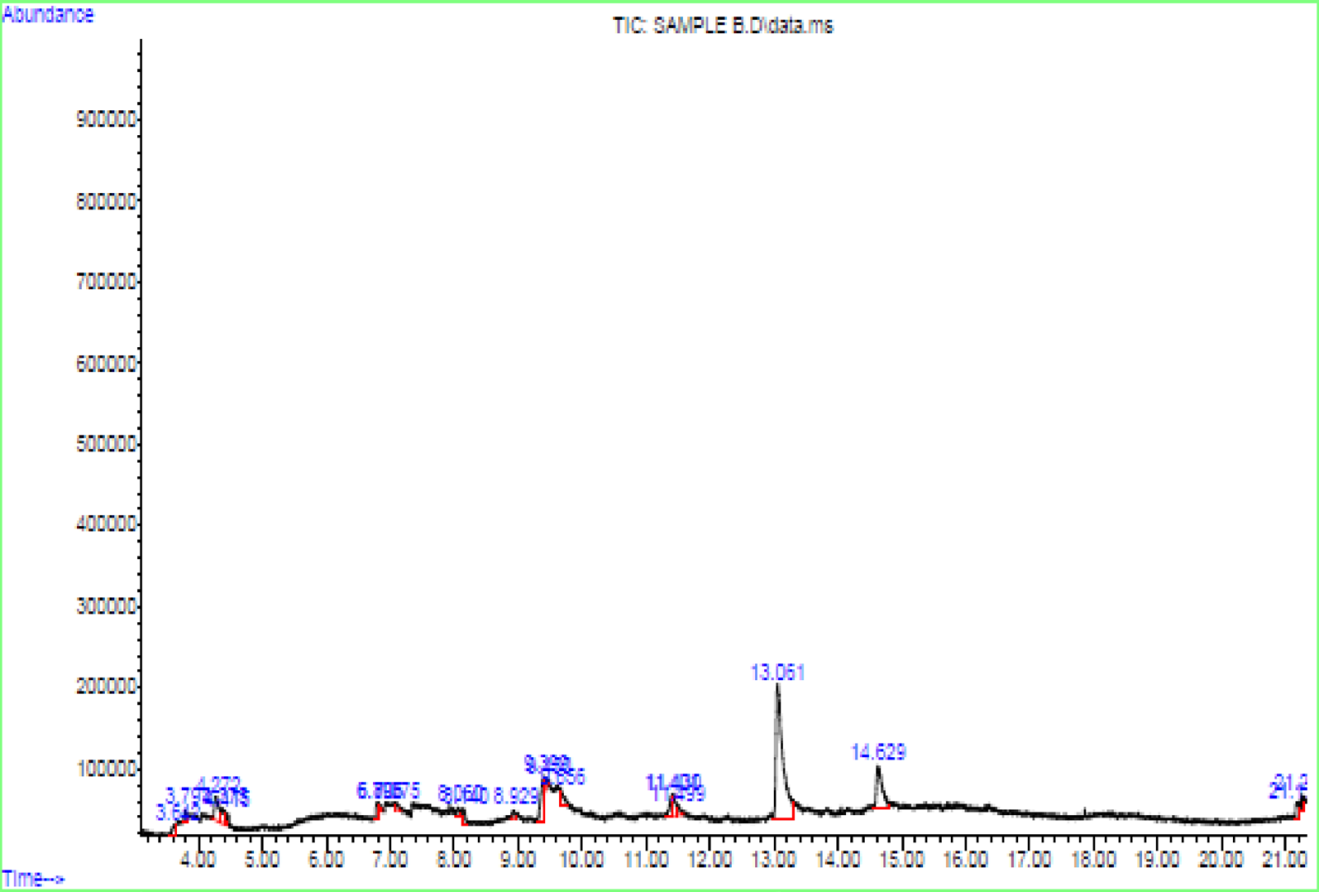
GC–MS chromatogram of *Azadirachta indica* (neem) leaf and root mixed extract

The GC–MS analysis of the neem leaf and root mixed extract, detailed in Fig. 1 and Table 3, identified a diverse array of bioactive compounds which including nitrogen-containing compounds, hydrocarbons, halogenated alkanes, heterocycles, fatty acid derivatives, and alcohols which matched the mass spectral data against the NIST database, confirming the presence of phytochemicals responsible for reducing and stabilising silver nanoparticles.

**Table 3:** GC–MS analysis of neem leaf and root mixed extract

RT	% Area	Compounds	Structures
3.74	1.82	Carboxylic acid	
3.81	1.19	Hexanol	
4.27	5.51	Fumaric acid	
4.38	1.78	Octanol	
6.81	0.97	Benzoic acid	
7.04	1.58	Myristic acid	
9.66	3.08	Decanoic acid	
14.6	11.64	Methyl stearate	

### Formation of silver nanoparticles

**Fig. 2:**
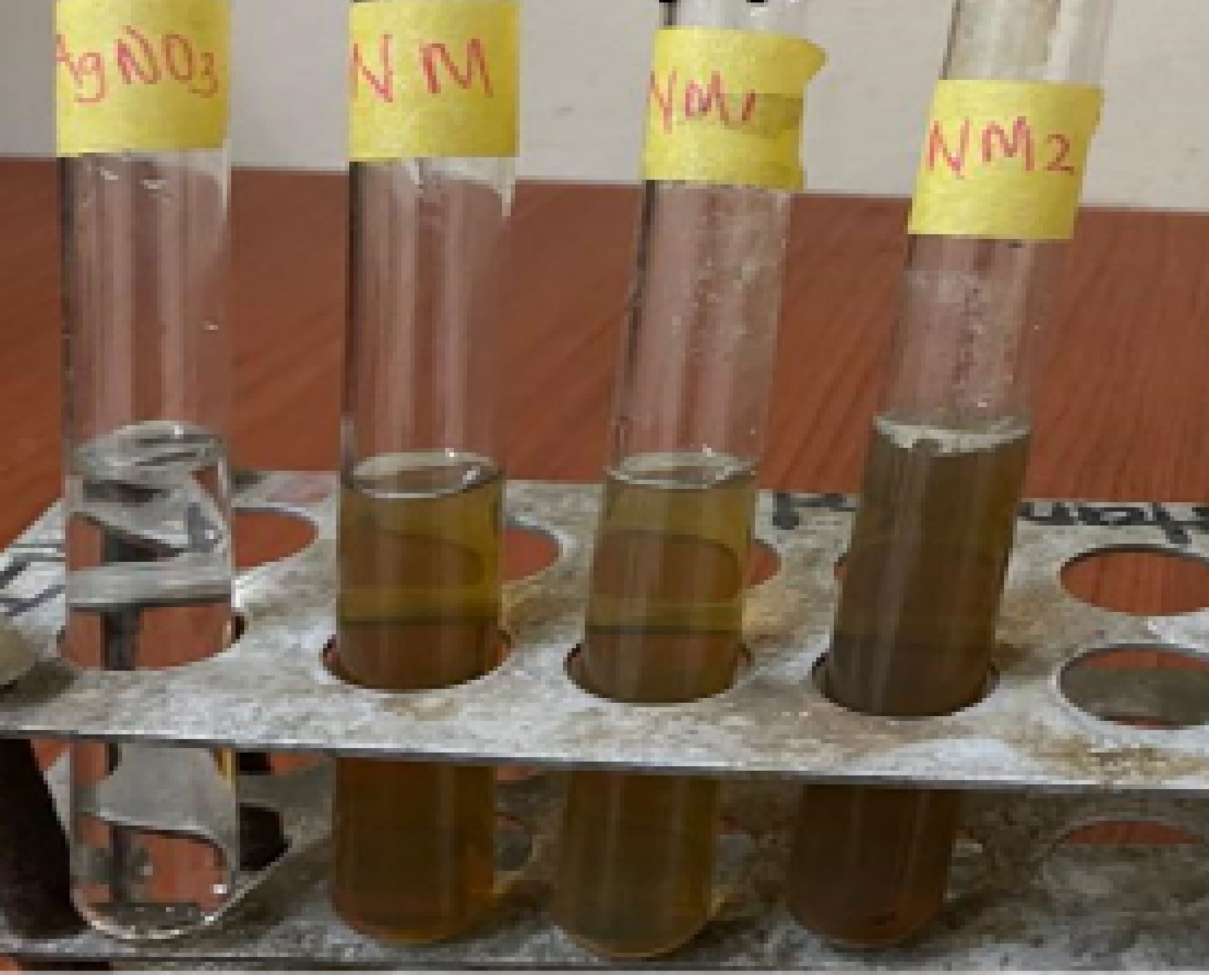
Visible colour change observed during the synthesis of silver nanoparticles using neem mixed extract (M). NM: neem mixed extract alone; NM + AgNO₃: reaction mixture at 0 h; NM1: after 1 h incubation; NM2: after 6 h incubation

The formation of AgNPs was observed within 60 min with a colour change from golden yellow to deep brown, providing visual confirmation of silver nanoparticle (AgNP) synthesis (Fig. 2a). The AgNPs displayed maximum UV–vis absorbance between 350 and 450 nm. In our previous study, it was observed that neem leaf extract, contains natural reducing agents like flavanones and terpenoids which play a vital role in reducing silver salts to AgNPs (Babatimehin et al. 2025a).

### Characterizations

**Fig. 3:**
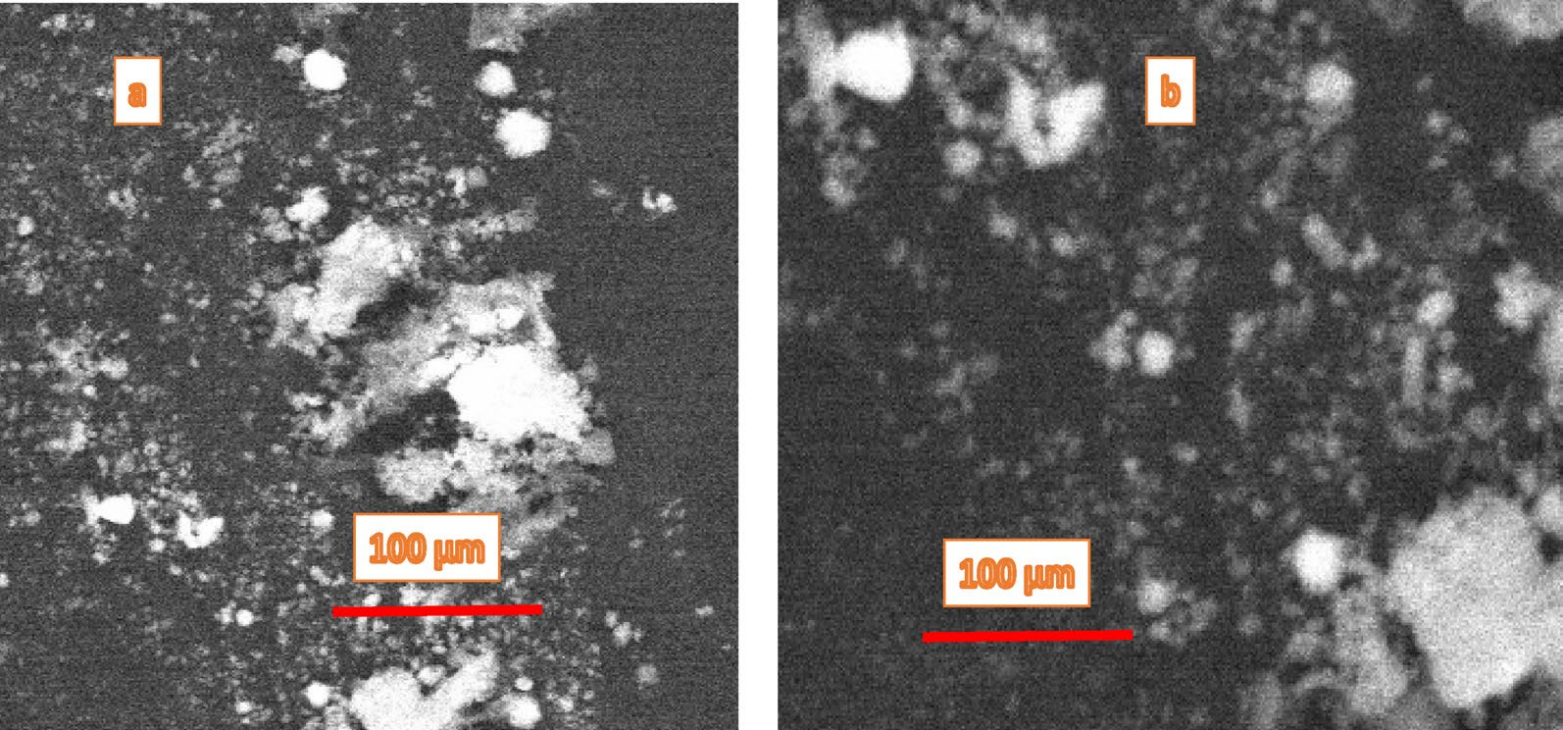
SEM micrographs of AgNPs synthesized using neem mixed extract at two extract concentrations: **a** 100 mg/mL and **b** 12.5 mg/mL

SEM assessment (Fig. 3) revealed agglomerated nanoparticles with round morphologies. For Fig. 3a**,** the particles appear more aggregated with visible clusters of different sizes, indicating a higher concentration of phytochemicals that enhanced rapid nucleation and particle growth. The larger bright regions suggest agglomerated silver nanoparticles, while the darker areas are background matrix or less dense regions. On the contrary, Fig. 3b shows a more dispersed morphology with relatively finer and smaller nanoparticles. The reduced clustering suggests that at this concentration of extract, the phytochemicals provided more controlled reduction and capping, causing smaller and more evenly distributed nanoparticles. This comparison indicates that the extract concentration significantly influences nucleation and stabilization of AgNPs.

**Fig. 4:**
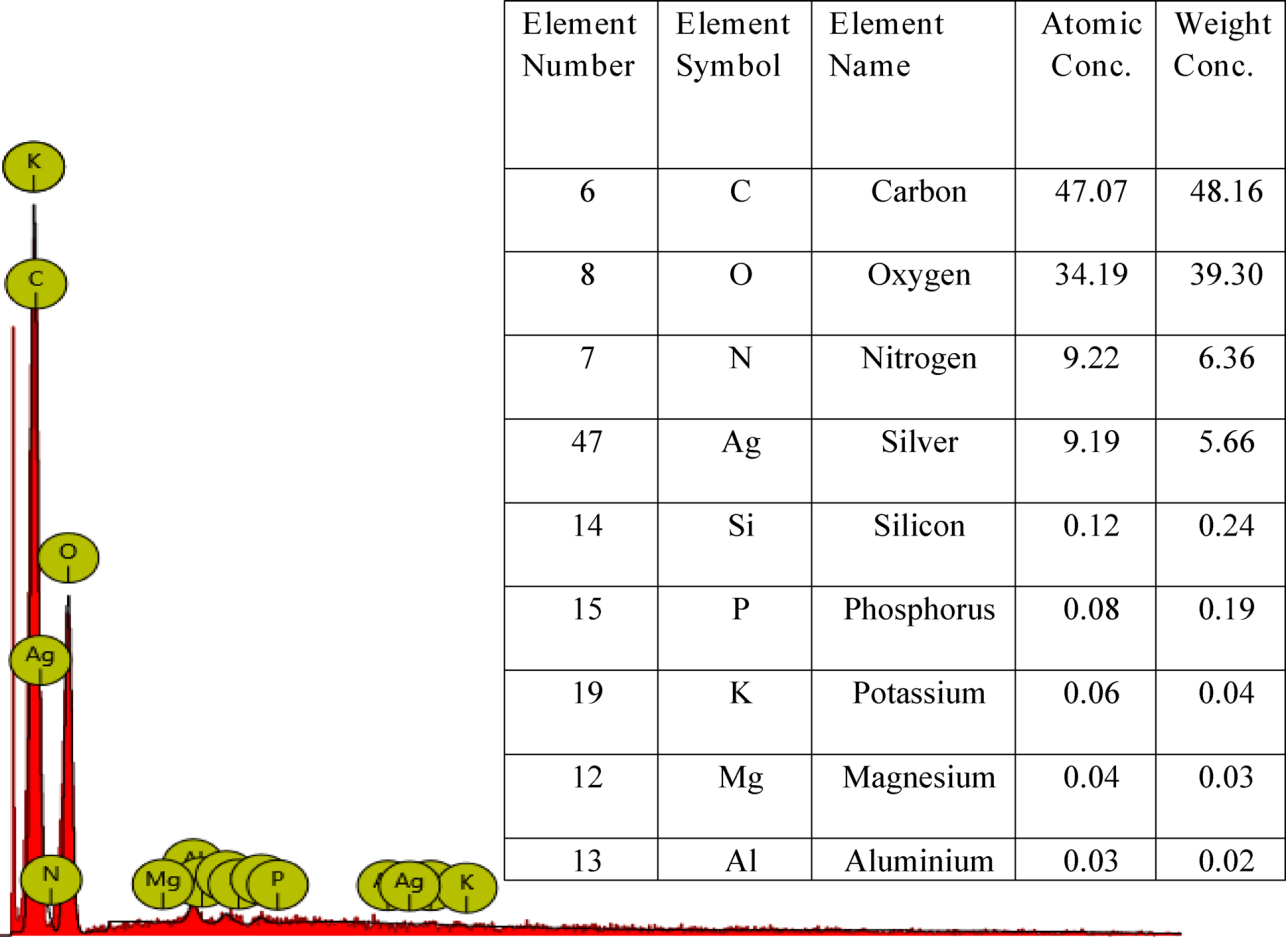
EDX analysis of the synthesized AgNPs at 50 mg/mL of the neem extract

The EDX spectrum of neem mixed extract–mediated AgNPs (Fig. 4) shows a strong signal for silver at around 3 keV, which is characteristic of metallic silver and confirms the successful reduction of Ag⁺ ions to Ag⁰ (Babatimehin et al. 2025b). Alongside silver, signals from C, Cl, Al, Na, Ca, Mg, Si, P, S, K, and Ti were also detected. These elements are associated with biomolecules present in the neem extracts that function as reducing and capping agents during nanoparticle synthesis. The high carbon content reflects the presence of organic phytochemicals (flavonoids, terpenoids, proteins, etc.), while elements such as Cl, Na, Mg, and Ca likely arise from natural salts and minerals in the plant matrix. Such phytochemical residues form a stabilizing corona around the nanoparticles, preventing agglomeration and contributing to their bioactivity. These findings are consistent with recent studies on plant-mediated synthesis of AgNPs, which reported carbon- and heteroatom-rich coatings together with silver as the core element (Bhat et al., 2019; Dhir et al., 2023) . The evidence from EDX analysis, therefore, not only confirms the successful production of neem-derived AgNPs but also supports the vital role of plant-derived metabolites in determining nanoparticle functionality, stability, and potential optical and biological applications.

**Fig. 5:**
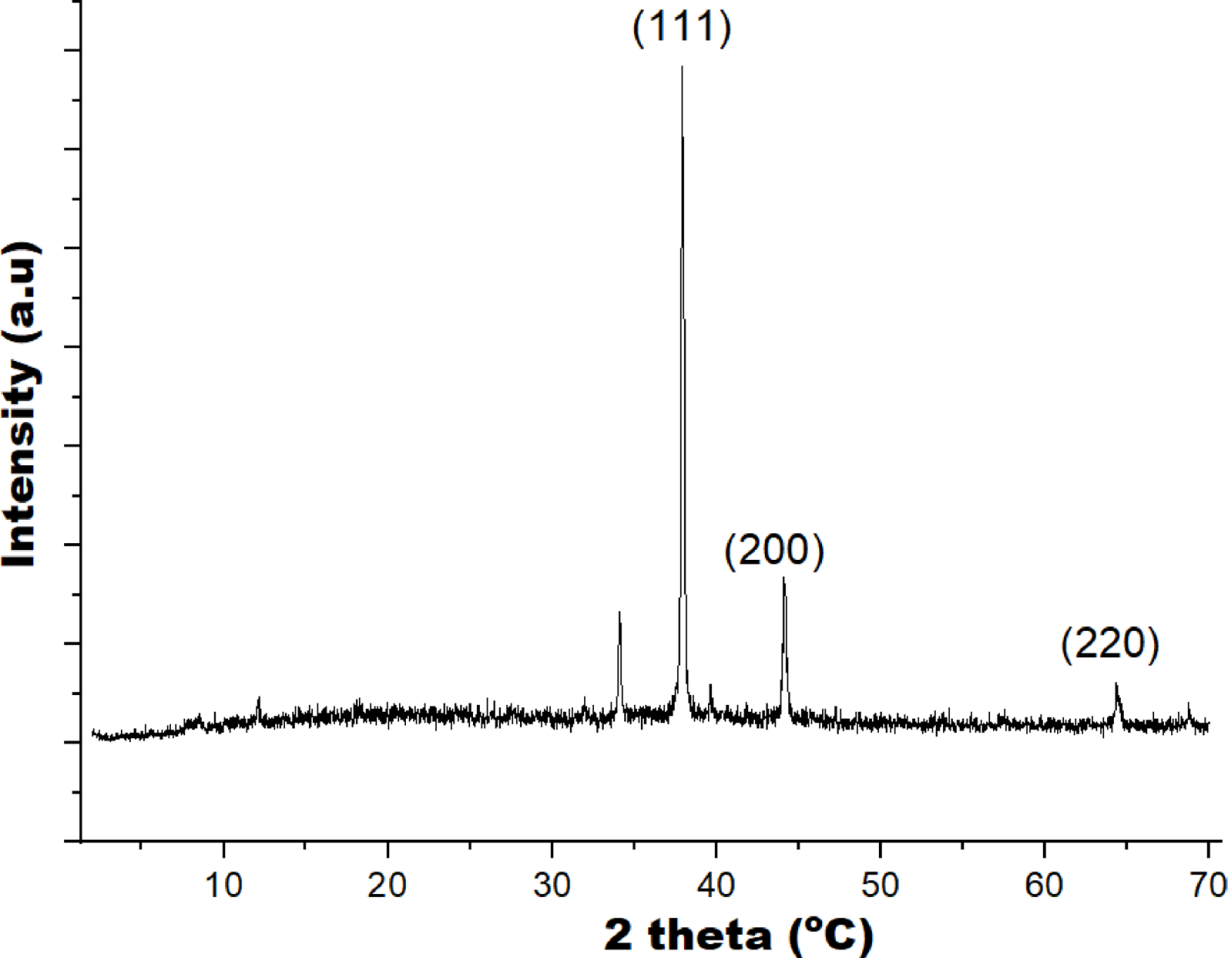
XRD diffraction pattern of AgNPs synthesized using 50 mg/mL neem mixed extract

Minor traces of other elements like P, Si, Mg, K, and Al are observed, which may originate from the plant matrix or impurities from the plant source. Their very low percentages (< 0.2%) suggest they do not significantly affect the nanoparticle composition but confirm the biomaterial origin of the reducing agents. The XRD study further validated the crystalline nature of the AgNPs, displaying distinct peaks at 38.8° (111), 45.0° (200), and 65.2° (220) (Babatimehin et al. 2025b), corresponding to a face-centred cubic structure of pure metallic silver (Fig. 5) (JCPDS card No. 04-0783). In addition to the sharp Bragg peaks, a broad shoulder between 30° and 35° (2θ) is clearly noticed, which is a feature property of plant-mediated, green-synthesized metal nanoparticles (Hosney et al. 2025; Gupta et al. 2025). This could be as a results of some factors like the presence of amorphous phytochemical residues from neem extracts that is rich in flavonoids, phenols, terpenoids, and reducing sugars which act as capping agents and generate a diffuse halo in the 28°–35° region; partially oxidized silver species such as Ag₂O, which are insufficient to form distinct crystalline peaks; poorly ordered phases or small amounts of non-crystalline, and nanocrystalline effects related to lattice strain and particle size (Hosney et al. 2025; Gupta et al. 2025). The crystallite size computed from the (111) reflection using the Scherrer equation was 54.31 nm, a range in which reduced coherence length and microstrain leads to peak broadening and further accentuate the observed shoulder.

**Fig. 6:**
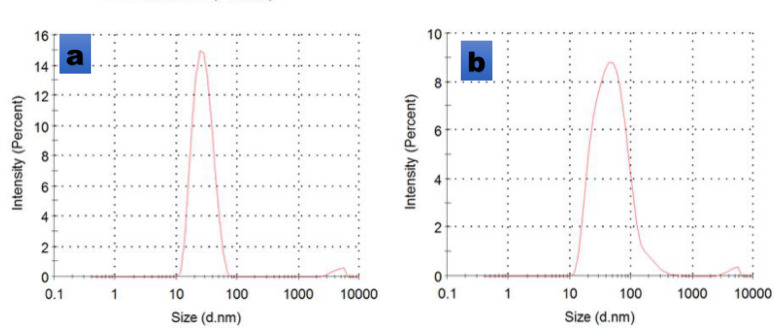
Particle size of synthesized silver nanoparticles at varying concentrations (a: 50 mg/mL and b: 100 mg/mL) of the neem leaf and root mixture

Figure 6 revealed the particle size investigation of the synthesized silver nanoparticles of the mixed extract and yielded AgNPs with an average size of 61.46 nm at 100 mg/mL, while lower concentrations (50 mg/mL) gave size ranges of 42–53.23 nm, respectively. The average particle size of the synthesized silver nanoparticles was observed to increase with higher concentrations of the neem leaf extract. Conversely, a narrower particle size distribution and a reduction in average diameter were achieved at lower extract concentrations, confirming the successful formation of nanosized materials under these conditions. Recent literature shows particle size measurements and zeta-potential determination are critical for evaluating the surface charge, size distribution, colloidal stability of nanoparticles which impact their long-term stability, aggregation behavior, and antimicrobial activity (Saki et al. 2025 and Shakib et al. 2024, Rodriguez-Loya et al. 2024).

**Fig. 7:**
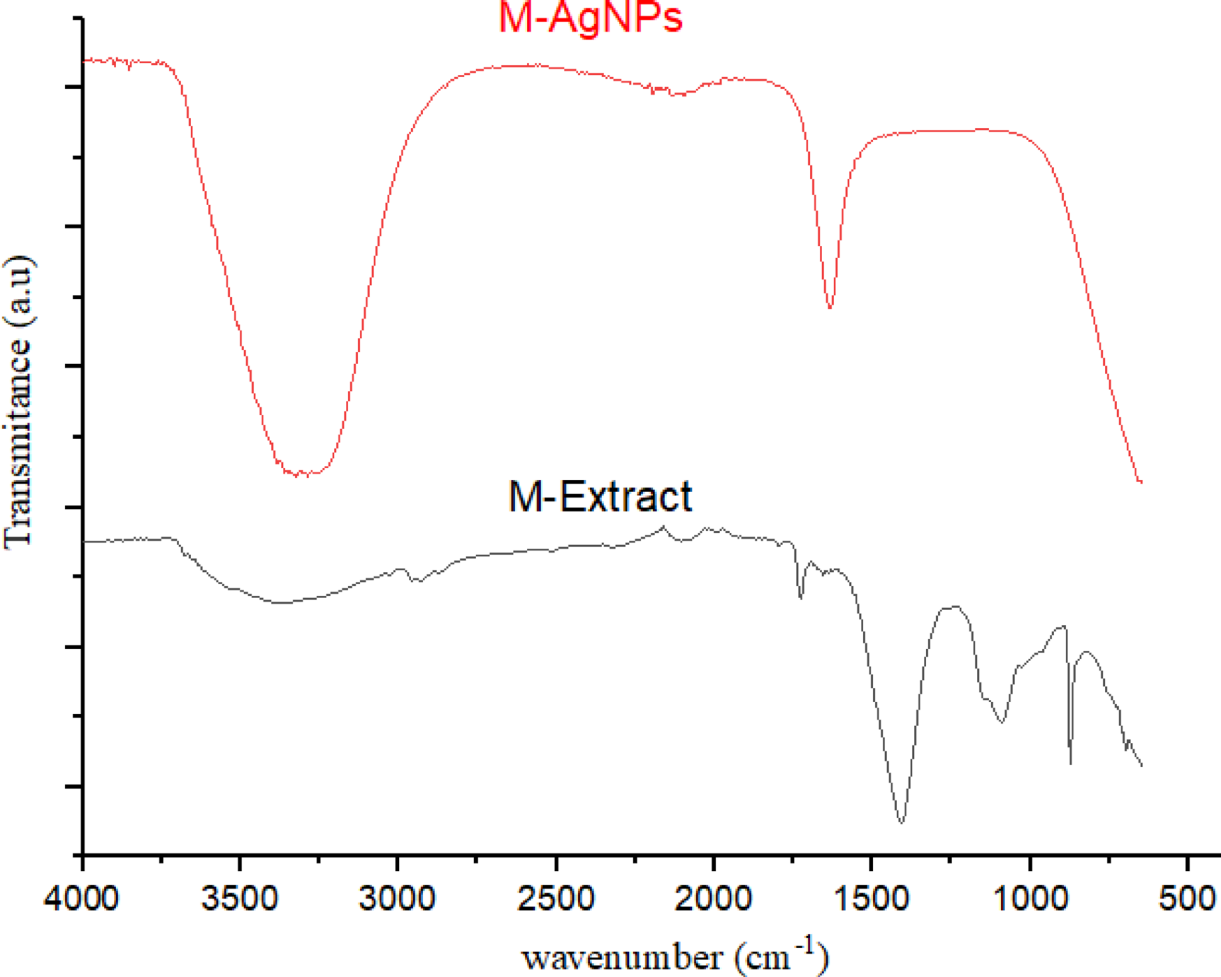
FT-IR measurement of (**a**) neem leaf and root mixed extract alone and (**b**) AgNPs synthesized from neem mixed plant extract

Fourier-transform infrared (FT-IR) spectroscopy was employed to identify the functional groups responsible for the reduction and stabilization of silver nanoparticles (AgNPs), confirming the involvement of various biomolecules from the neem leaf and root mixed extract (M-Extract) as shown in Fig. 7. The FT-IR spectrum of the neem leaf + root extract (M-Extract) displays broad O–H/N–H stretching of hydroxyl groups (phenols, flavonoids) and N–H stretching of amine/amide groups at ~ 3526–3284 cm^−1^ (Bhat et al. 2019; Alabdallah and Hasan 2021). Prominent alkane C-H stretching vibrations were detected at 2922 cm^−1^ and 2855 cm^−1^. The stretching attributed to—C=O band (carbonyl groups from flavonoids or proteins) and/or amide I (protein backbone) was seen at 1645 cm^−1^. The observed peaks at 1496 cm^−1^ is indication of amide II/N–H bending or aromatic C=C stretching (Bhat et al. 2019; Alabdallah and Hasan 2021). The observed peaks in the range of 1252–1025 cm^−1^ (notably 1081–1045 cm^−1^/1106 cm^−1^) are the –C–O stretching (ethers or alcohols), C–O–C and C–N stretching vibrations from glycosides and polysaccharides (Ansari et al. 2020; Ansari et al. 2023). The peak O–H/N–H observed in the extract at 3526–3284 cm^−1^ becomes broadened and shifts to 3384 cm^−1^ in the AgNPs spectrum, suggesting hydrogen-bonding and interaction of hydroxyl/amine groups with the Ag surface. The band attributed to C=O/amide I at 1645 cm^−1^ in the extract shifts to 1653 cm^−1^ with declined intensity in the AgNPs, consistent with carbonyl groups (from flavonoids/proteins) participating in the reduction of Ag⁺ and then capping the nanoparticle surface. The band at 1496 cm^−1^ (aromatic/amide II C=C) and the fingerprint peaks within 1252–1025 cm^−1^ region (C–O–C, C–O, C–N from glycosides and sugars) became weakened or disappear after nanoparticle formation, which is in supportive of the involvement (coordination/ oxidation) of these groups in the bioreduction and stabilization processes (Bhat et al. 2019; Alabdallah and Hasan 2021) In all, the spectral changes suggest that phenolic –OH, carbonyl/amide groups as well as sugar/polyol moieties in the neem extract actively reduce Ag⁺ to Ag⁰ and form a capping corona that stabilizes the nanoparticles (See Table 4). The flavonoid and phenolic compounds can contribute to the reducing power and capping activity via hydrogen bonding or π-metal interactions, carboxyl/carbonyl groups from acids, tannins, proteins may coordinate to silver surface, anchoring biomolecules. The amines/amides/proteins vibrations can bind through lone pairs on N, and stabilize via hydrogen bonding and steric hindrance, while the –C–O, C–C bands from carbohydrates/glycosides/polysaccharides can further contribute to capping and colloidal stabilization (Gholami et al. 2018; Pasieczna-Patkowska et al. 2025). In the study by Rahuman et al. 2022, when FT-IR was used to identify phytochemicals responsible for reduction and stabilization of AgNPs synthesized using biological extracts, it was established that plant-derived metabolites like proteins, phenolics, flavonoid, carboxylic acids, and aromatic rings remain bound to the AgNP surface after synthesis. In some studies, the peaks corresponding to –OH, N–H, C=O, C–O, and aromatic rings shift in position or intensity, and in some cases, completely disappear after nanoparticle formation indicating that these functional groups have bound to the nanoparticle surface (Pasieczna-Patkowska et al. 2025). Thus, the observed peak shifts in this present study likely reflect binding of phenolics/proteins/ flavonoids (through –OH, C=O, C–O, N–H) to the AgNP surface, which serves as a capping/stabilizing corona—preventing aggregation, enhancing stability, and influencing biological activity.

**Table 4:** Peaks assignment and changes in wavenumber of of neem leaf and root mixed extract alone and AgNPs synthesized from neem mixed plant extract

Extract Peak (cm^−1^)	AgNPs Peak (cm^−1^)	Δ (cm^−1^)	Functional group assignment	Observations
3526–3284	3384	+ 142/− 110	O–H/N–H	Peak broadening
2922, 2855	–		C–H	Complete disappearance
1645	1653	− 8	C=O/amide I	Shift and reduced intensity
1496	–	–	Amide II/aromatic C=C	Complete disappearance
1252–1025 (notably 1106, 1081–1045)	–	–	C–O/C–O–C/C–N	Complete disappearance

### Optimization studies

#### Effects of neem concentration

**Fig. 8:**
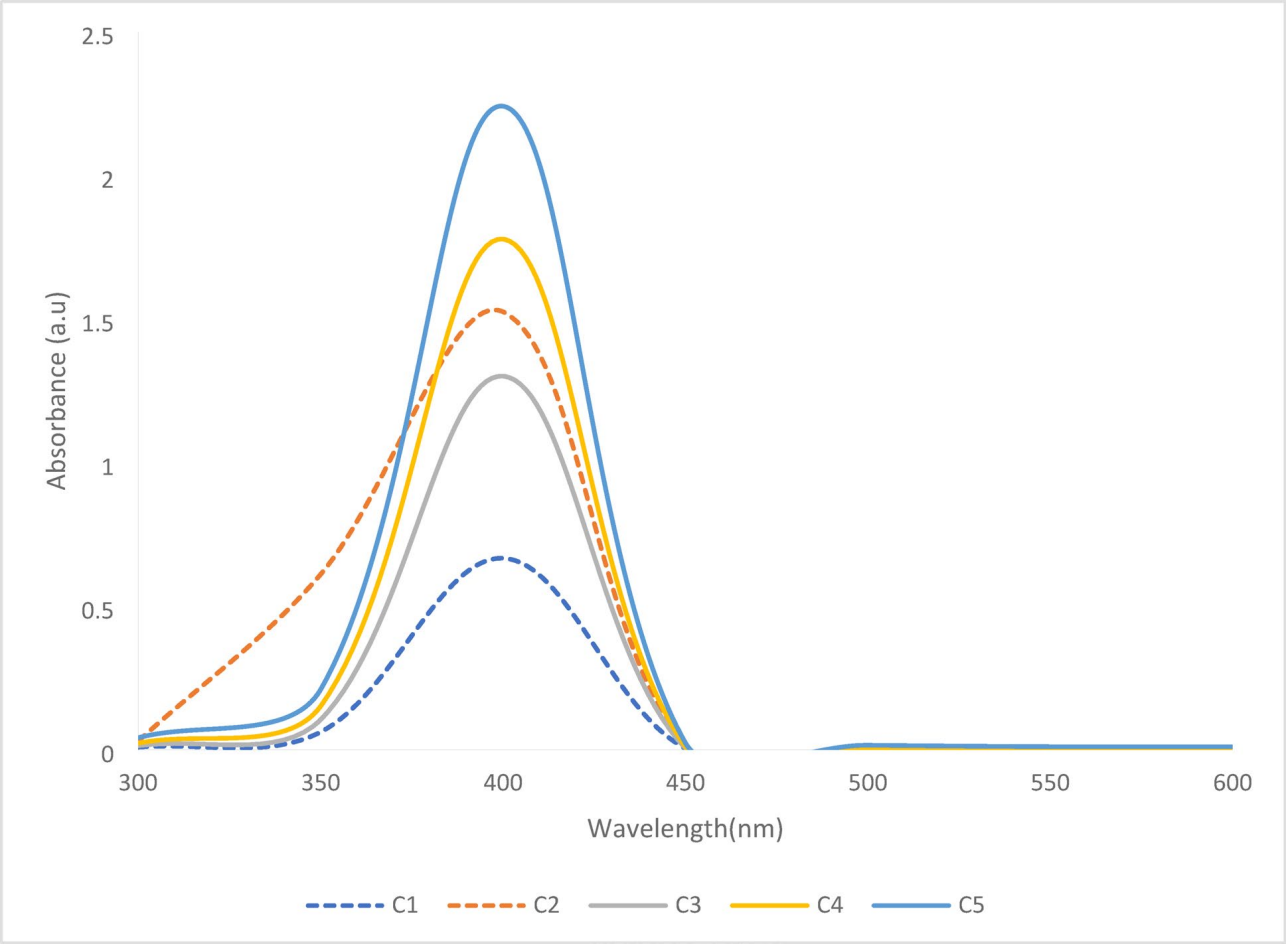
UV–vis spectra of synthesized neem leaf and root mixed AgNP at various concentrations of plant extract in 1 mM AgNO_3_ after a 6-h incubation (M = Neem mixed extract, C1 = 100, C2 = 50, C3 = 25, C4 = 12.5, C5 = 6.25)

The impact of extract concentration on the synthesis of the nanoparticles following a 6-h incubation in a 1 mM AgNO₃ solution (Fig. 8). All the spectra display a characteristic surface plasmon resonance (SPR) peak between 400 and 420 nm, confirming the formation of AgNPs after 6 h of incubation. It was observed that as the concentration of the plant extract increases (from C5: 6.25 to C1: 100), the absorbance intensity of the SPR peak also increases correspondingly, indicating enhanced nanoparticle formation likely due to greater availability of phytochemicals acting as reducing and stabilizing agents. Among all samples, C1 exhibited the highest absorbance, while C5 showed the lowest, establishing a direct relationship between extract concentration and AgNP synthesis efficiency. The shape and sharpness of the peaks also suggest that lower concentrations may produce more uniform nanoparticles, whereas higher concentrations could lead to broader size distributions or potential aggregation, as hinted by minor spectral fluctuations around 500 nm in the higher concentration samples. This finding is consistent with an earlier study by Ahmed and Mustafa (2020) where phytochemicals in plant extracts promote the reduction of Ag⁺ to Ag⁰, and the SPR peak becomes more prominent over time In this study, variations in extract concentration were found to impact the optical properties of the synthesized nanoparticles. An increase in the extract concentration offers a higher amount of reducing and stabilizing phytochemicals, which enables nucleation and limits particle growth. This subsequently leads to smaller, more uniformly capped nanoparticles, reflected by a slight blue shift and sharpening of the SPR peak (Babatimehin et al. 2025a). On the other hand, a lower extract concentration lessens the availability of these active phytochemicals, promoting the formation of larger particles or broader size distributions, which causes to a red shift or broadening of the SPR band (Ansari et al. 2023; Nosrati et al. 2025). These observations signify that the extract concentration plays a crucial role in modulating nanoparticle size and surface chemistry, thereby affecting the position and intensity of the SPR peak (Ansari et al. 2023; Nosrati et al. 2025). These trends align with our particle size (Fig. 6) and SEM observations (Fig. 3), confirming that extract concentration directly controls nanoparticle nucleation rate, size distribution, and the dielectric environment around the particles, all of which govern SPR behaviour.

### Effect of pH

**Fig. 9:**
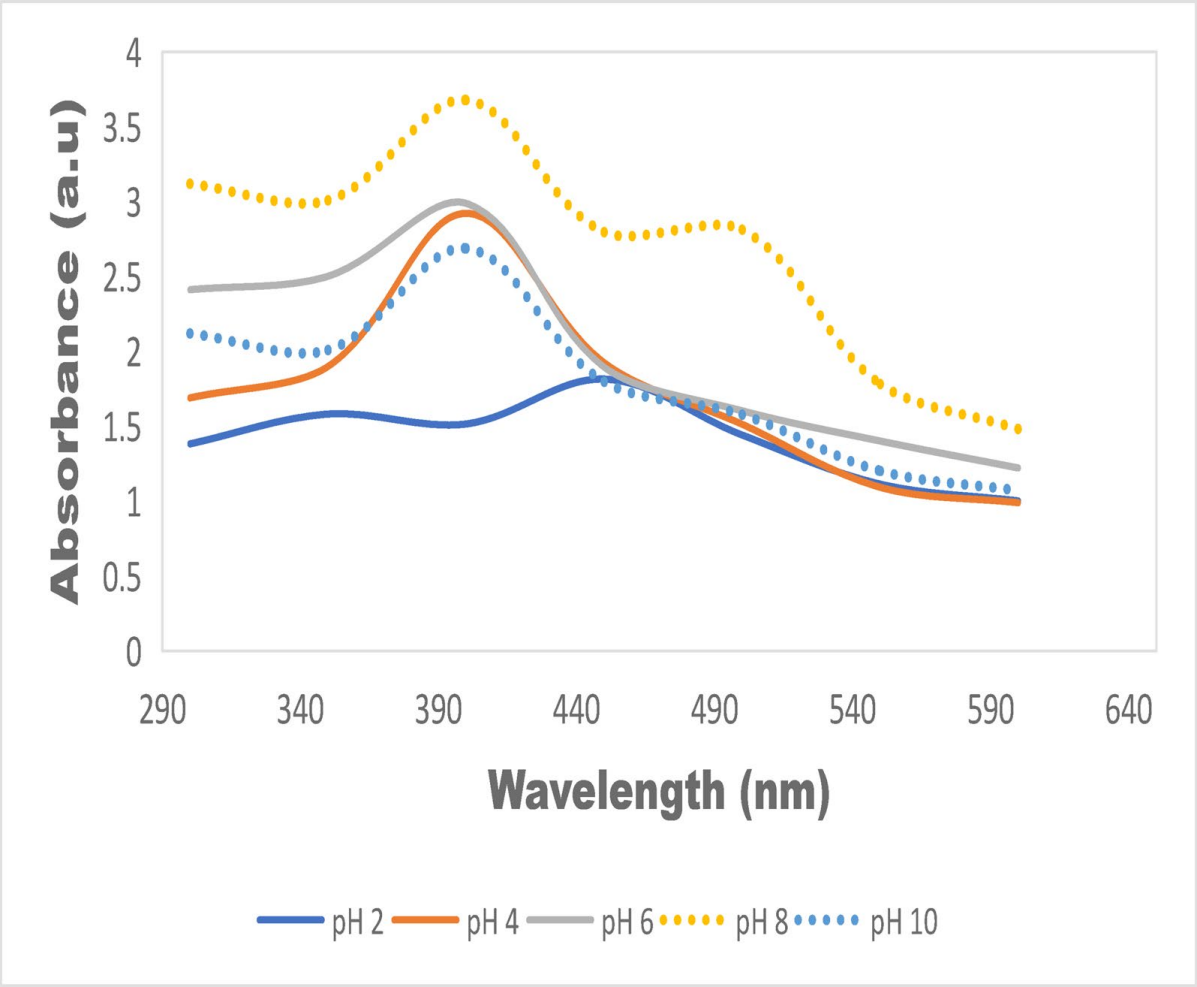
Absorption spectra of leaf and root mixed AgNPs (pH 2.0–10.0)

In this study (Fig. 9), it can be observed that the pH of the reaction medium exerted a strong influence on the formation, stability, and size of AgNPs synthesized using neem mixed extracts. Under highly acidic conditions (pH 2), weak and broad UV–Vis signals indicating limited formation of the nanoparticles. As pH rose toward neutrality and slightly alkaline (pH 6–8), the SPR peaks become sharper, more intense, and centered at ~ 390–420 nm, suggesting efficient nucleation into small, well-dispersed particles. At pH 8.0 specifically, the highest absorbance SPR profile is observed, suggesting optimum conditions. When the pH was further increased to 10.0, although absorbance remains high, there is noticeable broadening and red-shifting of the SPR band, consistent with larger average particle size, increased incipient or polydispersity aggregation. These observations agree well with recent reports by Ibrahim et al. (2024) where it was found that pH 10 is favorable towards AgNPs formation but noted increase in size for certain extract conditions; *Ixora coccinea* extract yielded smaller particles at alkaline pH against larger ones under acidic conditions; *Teucrium apollinis* showed optimal stability around pH 7–8; and studies using water hyacinth observed smallest sizes and highest yields around pH ~ 8.5. In another study by Zhang et al. (2025), it was observed that during the green synthesis of AgNPs using salicylic acid (SA), the SPR peak intensity gradually increased from pH 7 to 9, and reduced at pH 10, suggesting an optimum pH regime for nanoparticle formation. In the study on the impact of solvent and pH in synthesizing silver nanoparticles via green and chemical route by Das et al (2024), it was noted that at pH < 6, no significant LSPR peaks appear, but at neutral/basic pH, significant absorbance emerges, inferring that very acidic pH suppresses nanoparticle formation or gives unstable colloids. Thus, for neem leaf and root extract in this study, the optimal pH window appears to be ~ 6 to 8, with pH 8 giving the best balance of yield, size, and stability.

### Effect of temperature

**Fig. 10:**
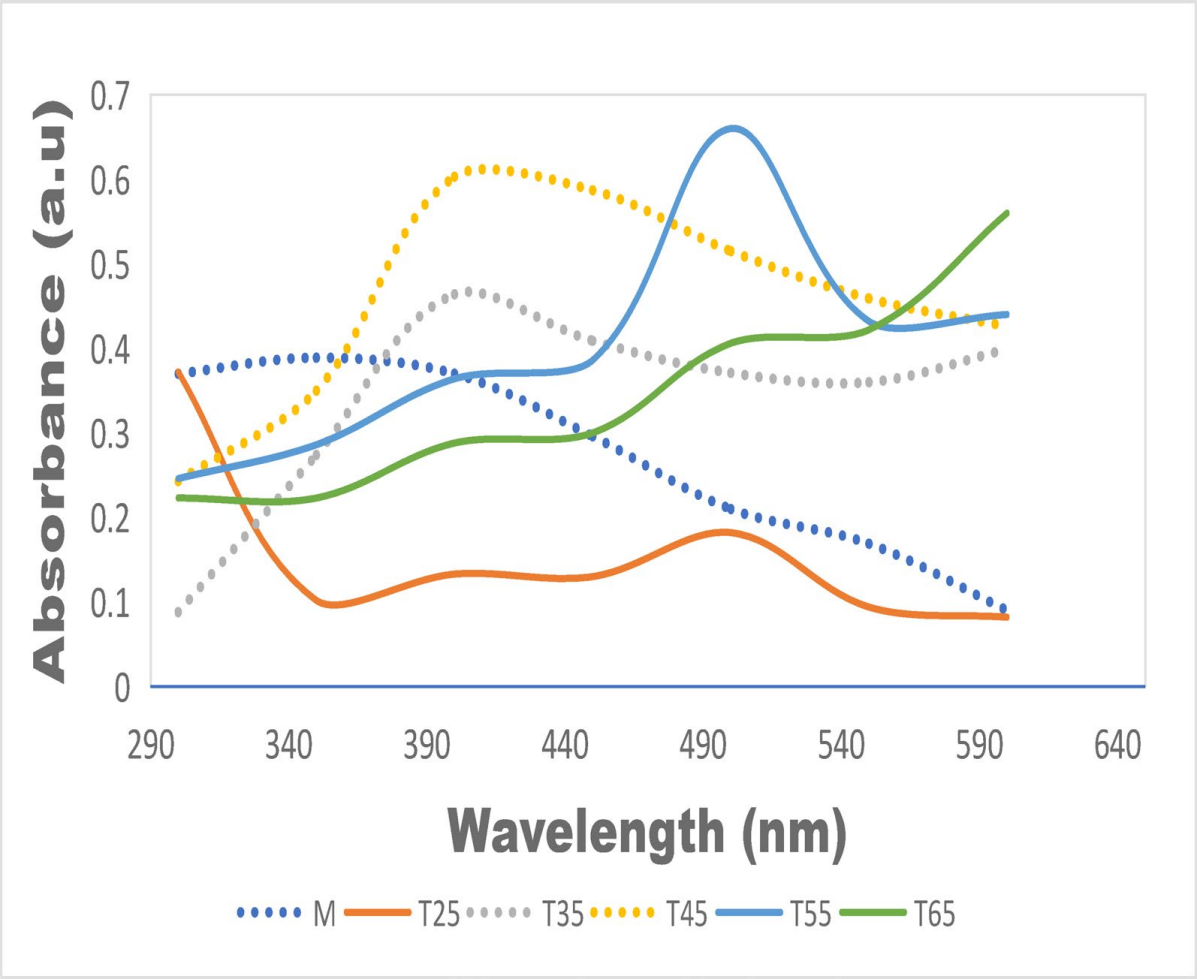
Absorption spectra of leaf and root mixed AgNPs at various Temperatures (M = neem mixture, T = temperature in °C)

Figure 10 shows the UV–Vis absorption spectra of AgNPs synthesized with neem leaf–root mixed extract at various reaction temperatures. No defined SPR peak appears between temperature of 25–35 °C, indicating negligible nanoparticle formation under these conditions. A pronounced SPR band was seen at 400–420 nm when the temperature rose to 45 °C and intensifies at 55 °C (λmax at 490–500 nm and Abs at 0.62–0.66 a.u.), suggesting efficient reduction of Ag⁺ and substantial nanoparticle formation. But at at 65 °C, the SPR band became broaden and red-shifts (~ 550–590 nm) consistent with increased average particle size and partial aggregation. These temperature-dependent trends with increasing SPR intensity up to an optimum temperature followed by red-shifting/broadening at higher temperatures thus agree with prior reports of plant-mediated AgNPs syntheses where temperature accelerates reduction but may reduce capping efficiency at very high temperatures as documented by Liaqat et al. (2022). The spectral evolution observed with temperature can be explained by the interplay of particle growth and nucleation. Elevated temperature enhances the rate of phytochemical-mediated electron transfer to Ag⁺, forming more nuclei and greater nanoparticle yields (higher SPR) (Mohd et al. 2024). Nevertheless, higher thermal energy can as well facilitates particle coalescence and can denature capping biomolecules, causing larger, less uniform particles and aggregation which could lead to red shift and broadening as observed in this present study at temperature of 65 °C. Sithara et al. (2017) observed that the SPR absorbance increases with temperature up to around 50 °C with maximum SPR at 424 nm, and thereafter declines at higher temperatures (> 50 °C) which was attributed to a likely degradation of biomolecule. Similarly, Gwada et al. (2025) noted that the SPR peaks were observed at around 431, 429, and 426 nm respectively; with increasing temperature, and that there was a blue shift, meaning smaller particles. They opined further that the peaks also tend to become narrow, consistent with more uniform growth, and improved nucleation at higher temperature (Gwada et al. (2025)). Furthermore, absorbance (SPR) was observed by Liaqat et al. (2022) to have increased with temperature; and that maximum absorbance was achieved at around 75 °C with peaks becoming more intense at that temperature for AgNPs with characteristic λ_max in the 410–430 nm region.

### Metal ions recognition and selectivity of neem mixed AgNPs

**Fig. 11:**
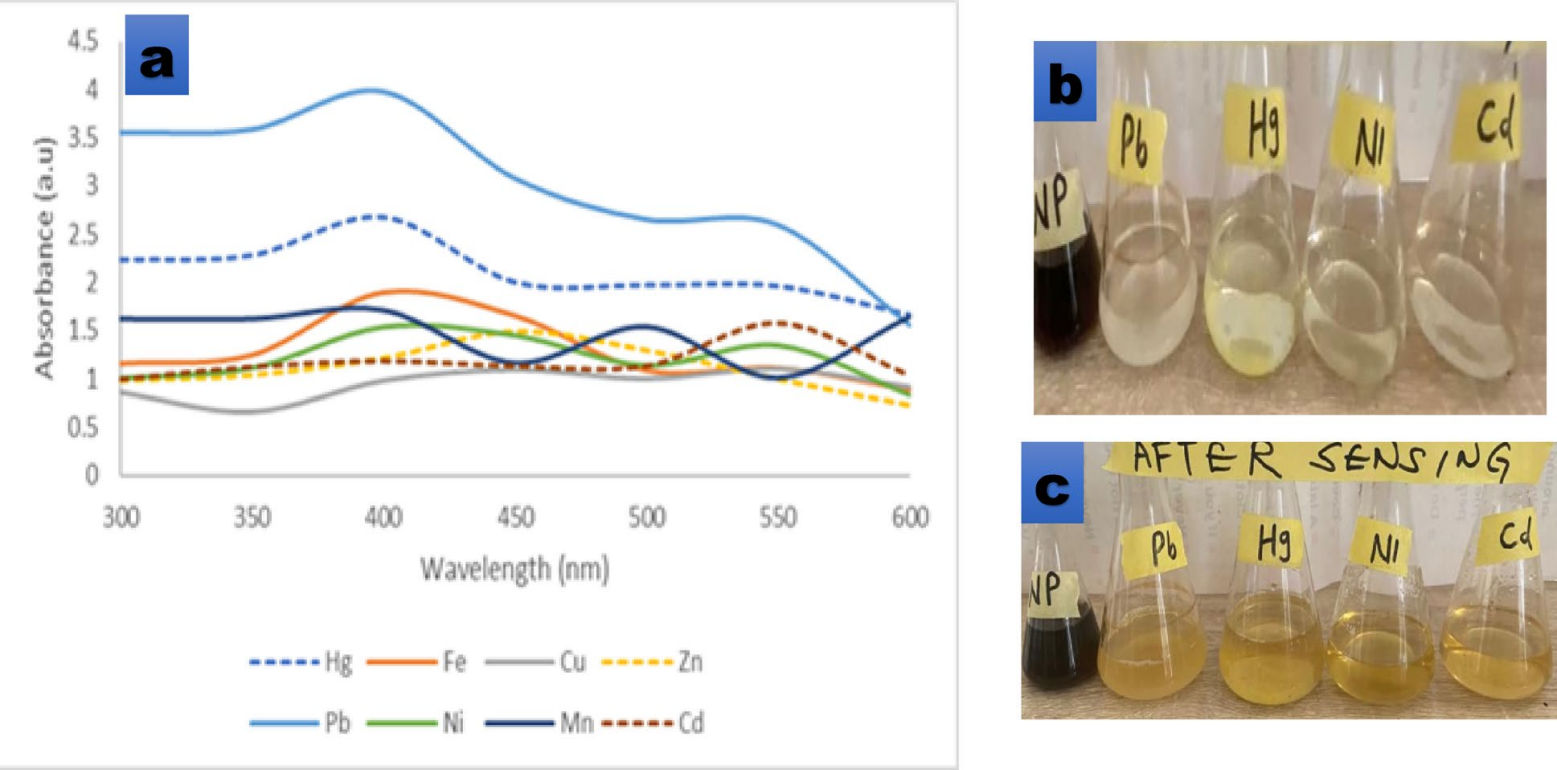
**a** UV–Vis spectra of AgNPs after interaction with various metal ions (Pb^2^⁺, Hg^2^⁺, Fe^3^⁺, Cu^2^⁺, Cd^2^⁺, Zn^2^⁺, Ni^2^⁺, Mn^2^⁺), **b** Colour of AgNP solution before metal-ion exposure, and **c** Visible colour changes after metal-ion exposure

The prepared neem leaf and root mixed AgNPs demonstrated significant optical responses upon exposure to various metal ions, as shown in Fig. 11a, b and c. The visible colour of the metals change from pale yellowish brown to darker shades after the introduction of Pb^2^⁺, Hg^2^⁺, Fe^3^⁺, Cu^2^⁺, Cd^2^⁺, Zn^2^⁺, Ni^2^⁺, and Mn^2^⁺ solutions demonstrates clear evidence of metal ion interaction with AgNPs. The UV–vis spectra (Fig. 11a) showed distinct absorbance variations across the 300–600 nm range, confirming metal-specific plasmonic perturbations. Among the sensed metal ions, Pb^2^⁺ exhibited the highest absorbance intensity (4.02 ± 0.13 a.u.) at around 400 nm, followed by Hg^2^⁺, which also showed strong enhancement in the 350–450 nm region. This indicates a high affinity of AgNPs towards Pb^2^⁺ and Hg^2^⁺, confirming their superior sensitivity for detection (better selectivity).

On the contrary, Ni^2^⁺ and Zn^2^⁺ displayed minimal spectral changes with smaller absorbance values, suggesting weaker interaction with the nanoparticle surface. The differential spectral shifts arise from variations in the charge transfer and binding affinity between the metal ions and the capping biomolecules of neem-mediated AgNPs. The selectivity observed align with earlier reports where it was noted that AgNPs obtained from sun-dried neem leaf extract selectively sensed Pb^2^⁺ (in addition to Hg^2^⁺) in micromolar range (Karthiga and Savarimuthu 2013). It has been explained that the nature of the phytochemical capping on AgNPs (like flavonoids, phenolics, terpenoids) plays a vital role in complexing or coordinating metal ions, thereby modulating local dielectric environment and shifting the localized surface plasmon resonance (LSPR) or causing aggregation (Pechyen et al. 2024). Furthermore, a recent neem-turmeric mediated AgNP synthesis showed stable plasmon peaks and potential for multifunctional application, suggesting the practical relevance of neem-based green AgNPs (Kumar et al. 2025). According to Chen et al. (2019), reactive groups such as -NH₂, -COOH, and -OH on AgNPs promote this crucial interaction between the synthesized AgNPs and the metal ions.

The ability of the green-synthesized AgNPs to sense metal ions is primarily due to the interaction between the phytochemical-functionalized nanoparticle surface and the metal ions with FT-IR investigations of plant-extract mediated AgNPs commonly reveal the presence of carbonyl, hydroxyl, and phenolic groups, which serve as electron-donating sites and facilitate reduction, capping, and stabilization of the nanoparticles (Bashir et al. 2025). When these metal ions are introduced, the surface groups can coordinate with the ions to form metal–ligand complexes, which partially neutralize surface charge and destabilize the colloid, leading to agglomeration. Such aggregation alters the colloidal stability and increases inter-particle interactions, causing changes in the local dielectric environment around the AgNPs (Ibrahim et al. 2024).

These structural changes produce measurable optical responses. Indeed, complexation and agglomeration are known to provoke a bathochromic shift (red-shift) or broadening of the surface plasmon resonance (SPR) peak due to enhanced plasmon coupling between adjacent particles, which modifies the collective oscillation of conduction electrons on the nanoparticle surface (Patel et al. 2023). In the present study, the SPR peak near 460 nm declined in intensity and broadened upon addition of metal ions—a behavior consistent with nanoparticle clustering and complex formation. Similar intensity suppression and broadening phenomena have been observed in several recent biosynthesized AgNP sensors (Ibrahim et al. 2024). These observations agree broadly with reports on bio-synthesized AgNP sensors. For example, several studies have shown that plant-derived AgNPs undergo visible color changes (yellow → brown, brown → dark brown/black) upon exposure to heavy metals—attributed to nanoparticle–ion complexation and aggregation (Chandraker et al. 2019; Patel et al. 2023). Shifts in SPR peaks have similarly been documented: red-shifts for ions such as Pb^2^⁺ and Cd^2^⁺, broadening of the plasmon band, or suppression of peak intensity—each linked to changes in nanoparticle size distribution and charge transfer processes between AgNPs and metal ions (Muhammad et al. 2023).

### Analytical performance

**Fig. 12:**
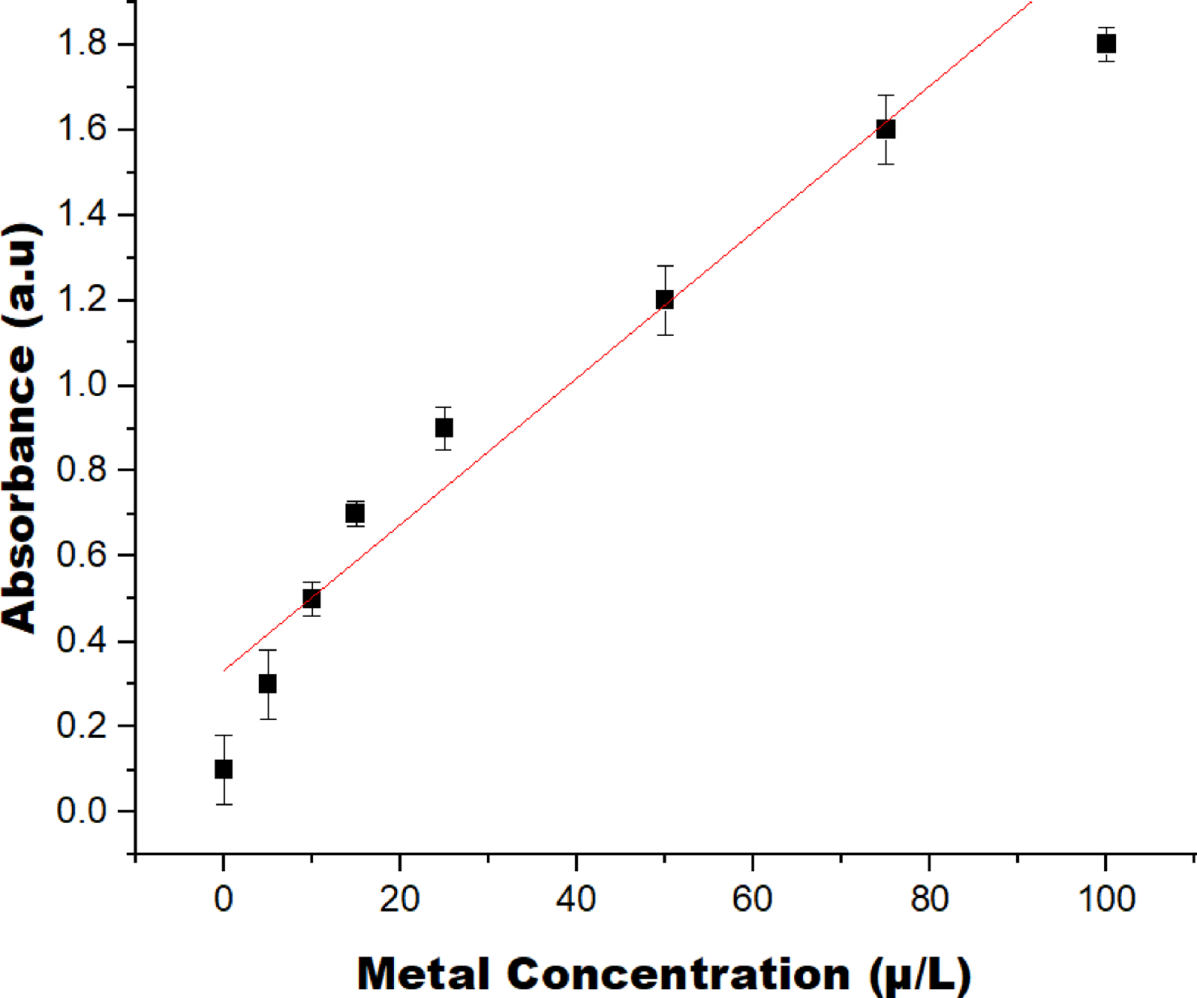
Calibration curve of colorimetric sensing of metal ions (0–100 µg/L)

**Fig. 13:**
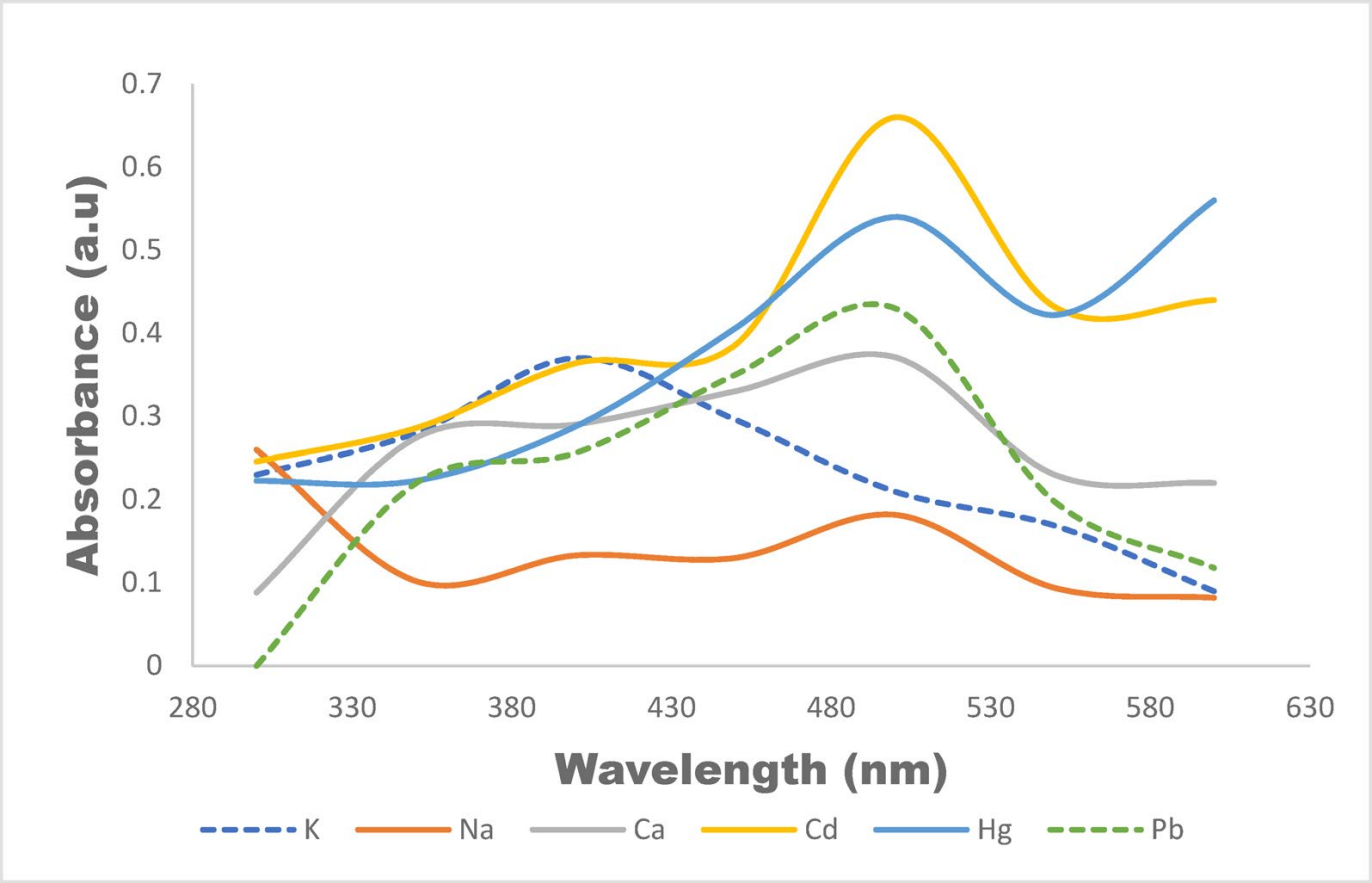
Selective absorbance of the AgNPs sensor toward Pb^2^⁺, Cd^2+^ and Hg ^2+^ in the presence of competing metal ions

The calibration curve obtained by plotting absorbance as a function of metal concentration in the range 0–100 µg L^−1^ is shown in Fig. 12. It was observed that the absorbance increases proportionally with concentration indicating a clear linear relationship between them. The computed correlation coefficient for the plot (R^2^) is 0.989. From the low-concentration region of the calibration curve, the standard deviation of the blank signal was estimated and this was subsequently used for the estimation of the limit of detection was estimated using the standard approach based on the standard deviation of the blank (σ) and the slope of the calibration curve (S), according to LOD = 3.3σ/S. Using the slope of 0.018 a.u. (µg L^−1^)^−1^, the LOD was estimated to be in the range of 5–6 ± 0.03 µg L^−1^. This value indicates the lowest concentration at which the metal can be reliably distinguished from the background signal. The limit of quantification was determined using LOQ = 10 σ/S. Addopting the same values for σ and S, the values of the LOQ was found within the range of 15–18 ± 0.25 µg L^−1^. Above this concentration, the calibration points indicate good alignment with the regression line and relatively small relative error bars, signifying acceptable accuracy and precision for quantitative analysis. The result is similar to the one reported by Sahu et al. (2024) where the same approach was used in colorimetric detection of As(III) using α-cyclodextrin-functionalized AgNPs, where linear calibration over a specified range enabled determination of an LOD of ~ 12.5 μg mL^−1^ and LOQ of ~ 38.9 μg mL^−1^. Similarly, Pb^2^⁺ sensing with AgNPs in the presence of dithizone as reported by Roto et al. (2019) exhibited a linear response between concentration and absorbance, yielding LOD and LOQ values of 0.64 ± 0.04 µg/L, and 2.1 ± 0.15 µg/L respectively. The absorbance response of the sensing system in the presence of competing ions, including K⁺, Na⁺, Ca2⁺, Hg2⁺, Cd2⁺, and Pb2⁺, to evaluate selectivity is shown in Fig. 13. The result show that K⁺, Na⁺, and Ca2⁺ exhibit weak and broad absorbance features with no distinct peaks across the studied wavelength range, signifying negligible interaction with the sensing probe. Their low and non-specific responses indicate that these common background ions do not significantly interfere with the sensing process. on the other hand, Pb2⁺, Hg2⁺ and Cd2⁺ produce a distinct and relatively intense absorbance band in the visible region, clearly differentiating themselves from the responses of K⁺, Na⁺, and Ca2⁺. These results affirm the good selectivity of the AgNPs sensor toward Pb2⁺ even in the presence of competing mono- and divalent metal ions.

### Antimicrobial screening synthesized AgNPs

**Fig. 14:**
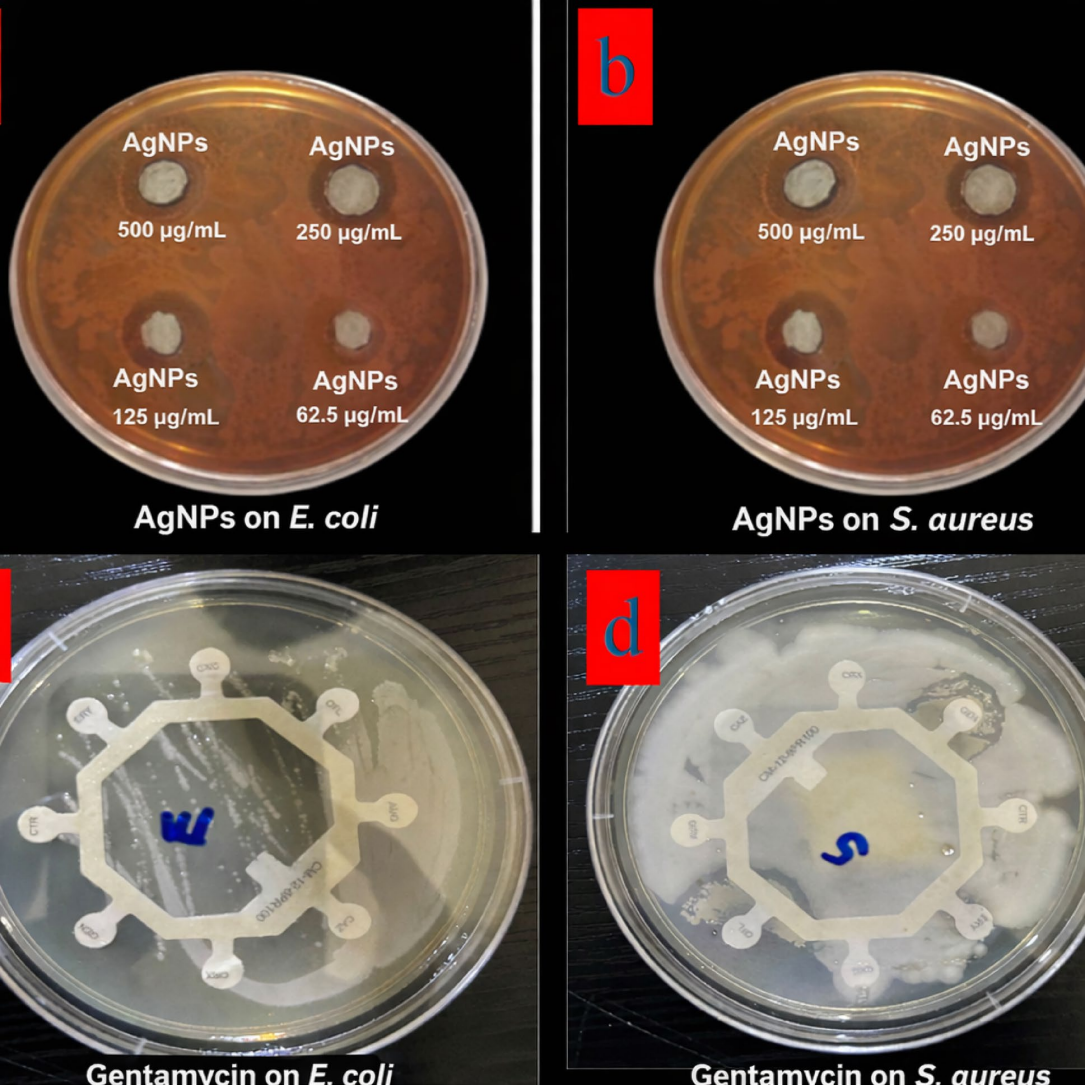
Antibiotic sensitivity assay showing labeled treatments and concentrations of: **a** AgNP treatments tested against *Escherichia coli*., **b** AgNP treatments against *Staphylococcus aureus*., **c** Gentamycin discs tested against *E. coli*., and **d** Gentamycin discs tested against *S. aureus*. All plates and individual wells/discs are labeled to indicate treatment type and applied concentration.

This study evaluated the antibacterial efficacy of silver nanoparticles (AgNPs) synthesized from *Azadirachta indica* (neem) mixed extract against gram-negative (*Escherichia coli*) and gram-positive (*Staphylococcus aureus*) pathogenic bacterial strains, using a well diffusion assay (Fig. 14a–d). Gentamycin was employed as a standard antibiotic control. The biosynthesized AgNPs demonstrated substantial antibacterial activity against both bacterial types, whereas the raw neem mixed extract alone showed no inhibitory effects. As summarized in Table 5, the zones of inhibition for the AgNPs measured 45 ± 0.21 mm for *E. coli* and 33 ± 0.13 mm for *S. aureus*, indicating strong antimicrobial potency. Table 6 further details the minimum inhibitory concentration (MIC), which was found to be 7 ± 0.12 µg/mL for *E. coli* and 5 ± 0.15 µg/mL for *S. aureus*. The enhanced antibacterial performance of AgNPs is attributed to their high surface area-to-volume ratio, which facilitates effective interaction with microbial cell membranes (Ershov and Ershov 2024). The neem-synthesized AgNPs in this work were effective against both gram-negative and gram-positive strains. This suggests that the surface chemistry and phytochemical capping of these biogenic nanoparticles may enhance their ability to overcome structural differences in bacterial cell walls.

**Table 5:** Zone of inhibitory zone assay of neem-synthesized AgNPs (n = 3)

Sample	Inhibitory zone	
	*E. coli*	*S. aureus*
Neem mixed extract	Nil	Nil
AgNO_3_	28 ± 0.13	17 ± 0.22
AgNPs	45 ± 0.21	33 ± 0.13
Gentamycin	22 ± 0.23	14 ± 0.14

**Table 6:** Minimum inhibitory concentration of neem-synthesized AgNPs (n = 3)

	Minimum inhibition concentration (µg/mL)
*AgNO*_*3*_	2 ± 0.23
*E. coli*	7 ± 0.12
*S. Aureus*	5 ± 0.15
Gentamycin	8 ± 0.31

These findings align with recent reports, in which AgNPs derived from some plants extracts exhibited antimicrobial activity against both gram-positive and gram-negative bacteria (Kemala et al. 2024; Gwada et al. 2025). However, the observed broad-spectrum efficacy in this study highlights the potential of neem-based AgNPs as versatile and effective antimicrobial agents, warranting further investigation into their clinical applications. The ability of neem-mediated AgNPs to suppress fungal or bacterial growth could be attributed to the synergetic effect of phytochemicals and silver ions disrupting cell membranes, generating reactive oxygen species, and interfering with cellular machinery (Khalifa et al. 2025).

### Cytotoxicity evaluation (MTT assay)

**Fig. 15:**
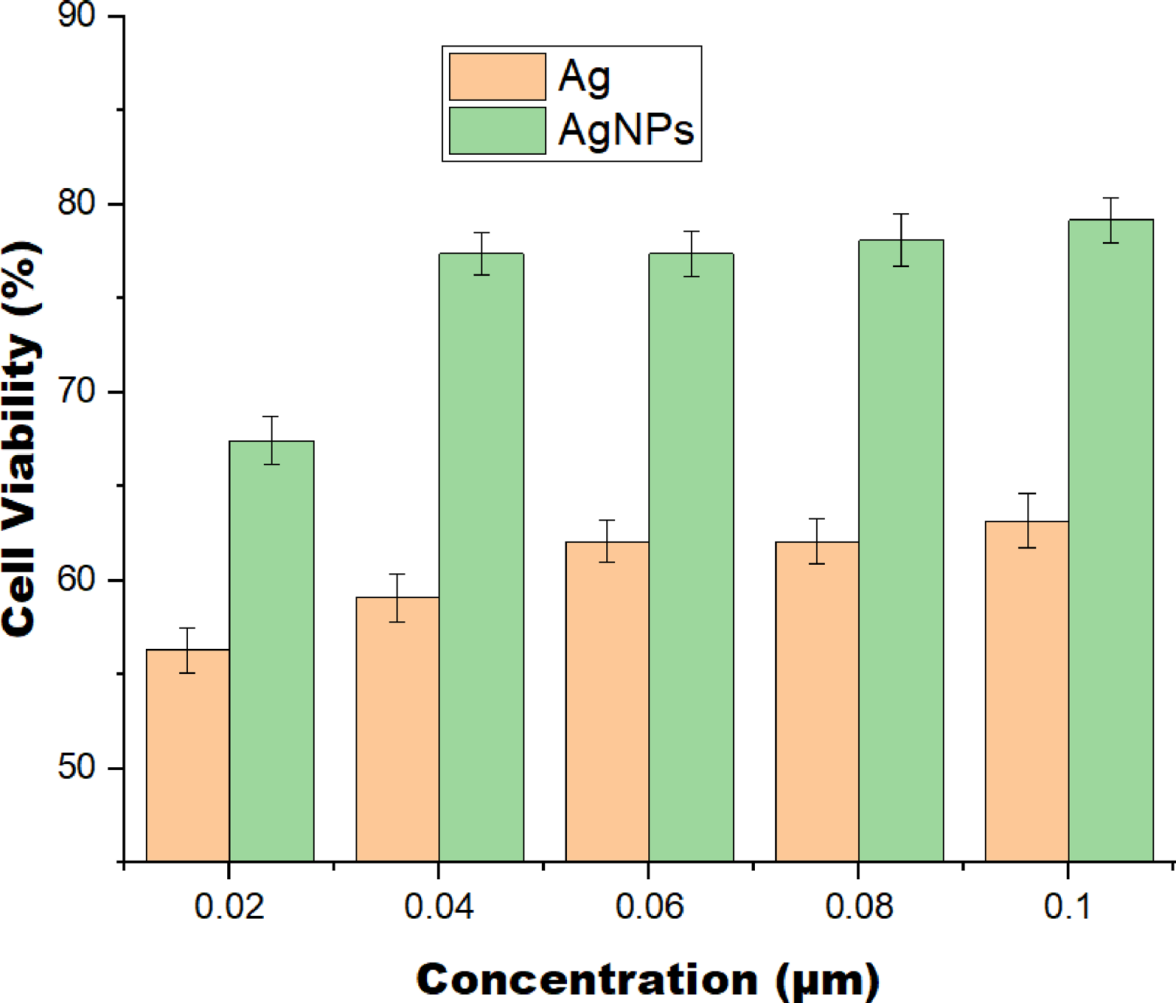
Cell viability study using synthesized AgNPs

The cytocompatibility of Ag and AgNPs was assessed using the MTT assay, and the results are presented in Fig. 15. Across all tested concentrations (0.02–0.1 μm), AgNP-treated cells consistently exhibited higher viability compared to those exposed to Ag. While Ag reduced viability to approximately 56–64%, AgNPs maintained higher levels, ranging from about 67–79%. This clear difference suggests that AgNPs are inherently more biocompatible than Ag under the tested conditions, likely due to differences in surface properties, interactions with cellular components or ion-release behaviorx. Interestingly, both materials showed a slight increase in viability with increasing concentration, indicating that the concentrations employed were below the cytotoxic threshold for NIH3T3 cells and may even promote mild metabolic activity. The findings reveal that AgNPs exert markedly lower cytotoxic effects than Ag and therefore represent a more favorable form for biomedical applications requiring enhanced cell compatibility.

Similar to previous studies examining AgNP biocompatibility (Meher et al. 2024; Eker et al. 2024), the present work reveals that AgNPs demonstrated considerably higher cell viability than Ag across all tested concentrations. This trend corroborates the findings by Barbalinardo et al. (2025), who showed that nanoparticle surface functionalization can significantly reduce cytotoxicity. Furthermore, Hosny et al. (2025) stressed that controlled ion release and nanoscale surface features contribute to the improved cytocompatibility noticed in AgNP systems.

### Novelty and comparison with prior neem-based AgNP studies

Biosynthesis of AgNPs using neem extracts has been widely reported, with most studies—including our previous leaf-only AgNP work (Babatimehin et al. 2025a) which focused on individual plant parts. On the other hand, this present study employs a combined leaf + root extract**,** which provides a richer and more diverse pool of phytochemicals for silver reduction and capping. The broader functional-group composition leads to more pronounced FT-IR shifts, broader and pronounced fingerprint features, and stronger biomolecule–Ag interactions than those reported in leaf-only systems. The summary of the key distinctions between this study, Babatimehin et al. (2025a, b), and other neem-mediated AgNP as documented in literature are presented in Table 7.

**Table 7:** Comparison of the present work with prior neem-based AgNP works

Feature/Criterion	Present study (leaf + root extract)	Babatimehin et al. (2025a, b) (leaf extract only)	Typical neem-AgNP studies in literature
Plant material used	Combined leaf + root extract	Leaf extract only	Mostly leaf-only, occasionally bark/seed
Phytochemical diversity	Higher (root adds terpenoids, alkaloids, glycosides)	Moderate (phenolics, flavonoids)	Moderate; usually single-tissue
Reduction/capping behavior (FTIR)	Stronger shifts in O–H, C=O, C–O (broader functional involvement)	Narrower FTIR shifts	Similar to leaf-extract patterns
Characterization techniques used	UV–Vis, FTIR, SEM	UV–Vis, FTIR, SEM	Usually UV–Vis + FTIR; some include SEM
Application tested	Heavy metal sensing and antibacterial study	Heavy metal sensing and antibacterial study	Mostly antibacterial or dye degradation
Novel contribution	Mixed-extract synthesis + functional-group analysis + dual adsorptions	Leaf-only AgNP synthesis	Mostly single-part plant extracts; rare mixed extracts

## Conclusions

This work demonstrates a sustainable path for synthesizing silver nanoparticles using mixed neem leaf–root extracts, highlighting how naturally occurring phytochemicals can simultaneously drive reduction, capping, and functional enhancement of biogenic nanomaterials. By integrating systematic optimization with comprehensive characterization, the study founds a clear link between reaction conditions, extract composition, and nanoparticle performance. A key innovation of this work is the dual-functional platform achieved from a single green-synthesis process: the resulting AgNPs demonstrate both robust antimicrobial property and selective responsiveness toward environmentally relevant heavy metals. This combined therapeutic- sensing potential underscores the value of neem-derived nanomaterials as cost-effective alternatives to conventional chemical syntheses and instrumental detection systems. In all, the study advances the understanding of plant-mediated nanoparticle formation and provides a practical, eco-friendly framework for developing multifunctional nanomaterials for biological and environmental monitoring applications.

## Data Availability

The data that support the findings of this study are available from the corresponding author upon reasonable request.
